# Supplementary material for: Signals from the niche promote distinct modes of translation initiation to control stem cell differentiation and renewal in the Drosophila testis
Source: PLoS Biol. 2025 Mar 11;23(3):e3003049. doi: 10.1371/journal.pbio.3003049 (PMC12136000; doi:10.1371/journal.pbio.3003049)
Supplement: S1 Table — Percent of testes with ectopic CySCs (assessed as Zfh1-positive, Eya-negative cells at least 3 cell diameters from the hub), or fewer than 10 CySCs near the hub. Note that some knockdowns with ectopic CySCs also occasionally led to a complete absence of somatic cells, so when ectopic cells were observed, the reduction of CySCs near the hub was not assessed. (DOCX) [file pbio.3003049.s001.docx]

**Table S1 – Summary of initiation factor screen.**

Percent of testes with ectopic CySCs (assessed as Zfh1-positive, Eya-negative cells at least 3 cell diameters from the hub), or fewer than 10 CySCs near the hub. Note that some knockdowns with ectopic CySCs also occasionally led to a complete absence of somatic cells, so when ectopic cells were observed, the reduction of CySCs near the hub was not assessed.

| **Knock-down** | **Stock number** | **% testes with ≤10 CySCs** | **Ectopic CySCs (%testes)** | **Number of testes examined** |
| --- | --- | --- | --- | --- |
| Control | NA | 0 | 0 | 26 |
| eIF4G1 | VDRC 17003 | 95 | 0 | 19 |
| eIF4A | VDRC 42202 | 100 | 0 | 17 |
| eIF4A | VDRC 100310 | 91 | 0 | 22 |
| eIF4E1 | VDRC 17581 | 27 | 0 | 22 |
| eIF4E1 | VDRC 7800 | 77 | 0 | 13 |
| eIF4E3 | VDRC 34210 | 0 | 0 | 21 |
| eIF4E4 | VDRC 107595 | 0 | 0 | 30 |
| eIF4E6 | VDRC 17580 | NA | 20 | 15 |
| eIF4H1 | VDRC 34301 | 0 | 0 | 19 |
| eIF4H2 | VDRC 102825 | 0 | 0 | 16 |
| eIF4B | VDRC 31364 | 0 | 0 | 15 |
| eIF3a | VDRC 28140 | NA | 80 | 15 |
| eIF3b | BDSC 32880 | NA | 86 | 14 |
| eIF3b | VDRC 107829 | NA | 82 | 31 |
| eIF3c | VDRC 26667 | NA | 40 | 5 |
| eIF3d1 | NIG 073-09 | 100 | 0 | 17 |
| eIF3d2 | VDRC 104342 | 0 | 0 | 16 |
| eIF3e | VDRC 27032 | NA | 41 | 22 |
| eIF3f | VDRC 101465 | NA | 50 | 22 |
| eIF3g | VDRC 28937 | NA | 65 | 23 |
| eIF3h | VDRC 106189 | NA | 78 | 37 |
| eIF3i | VDRC 27032 | NA | 90 | 20 |
| eIF2α | VDRC 7799 | NA | 75 | 40 |
| eIF2γ | VDRC 39377 | NA | 60 | 15 |
| eIF1 | VDRC 29216 | NA | 16 | 18 |
| eIF1A | VDRC 26022 | NA | 41 | 22 |
| eIF6 | VDRC 108094 | NA | 35 | 17 |
| pAbp | VDRC 22007 | NA | 96 | 25 |
| eIF2D | BDSC 33995 | 0 | 0 | 16 |
| DENR | VDRC 28105 | NA | 5 | 18 |
| NAT1 | BDSC 27302 | 0 | 0 | 23 |
