# Supplementary material for: Signals from the niche promote distinct modes of translation initiation to control stem cell differentiation and renewal in the Drosophila testis
Source: PLoS Biol. 2025 Mar 11;23(3):e3003049. doi: 10.1371/journal.pbio.3003049 (PMC12136000; doi:10.1371/journal.pbio.3003049)
Supplement: S2 Table — (DOCX) [file pbio.3003049.s002.docx]

**Table S2: list of genotypes shown in each figure.**

| **Figure panel** | **Genotype** |
| --- | --- |
| 1C | *Oregon-R* |
| 1D | *w/Y;; FRT^82B^ Stat92E^397^, ca, e/ +* |
| 1E | *w/Y;; FRT^82B^ Stat92E^397^, ca, e/ mwh red Stat92E^Frankenstein^ e* |
| 1F | *w/Y; tj-Gal4/+; tub>Gal80^ts^/+* |
| 1G | *w/Y; tj-Gal4/+; tub>Gal80^ts^/UAS-upd* |
| 1H | As in Fig. 1C |
| 1I | “control” as in Fig. 1D, “*Stat92E^F^/Stat92E^397^”* as in Fig. 1E |
| 1J | “control” as in Fig. 1F, “*tj^ts^>upd”* as in Fig. 1G |
| 2B | *w/Y; tj-Gal4/+; tub>Gal80^ts^/+* |
| 2C | *w/Y; tj-Gal4/+; tub>Gal80^ts^/UAS-eIF4A RNAi* |
| 2D | *w/Y; tj-Gal4/UAS-eIF4G1 RNAi; tub>Gal80^ts^/+* |
| 2E | *w/Y; tj-Gal4/+; tub>Gal80^ts^/eIF4E1 RNAi* |
| 2F | *w/Y; tj-Gal4/UAS-eIF3h RNAi; tub>Gal80^ts^/+* |
| 2G | *w/Y; tj-Gal4/UAS-eIF1A RNAi; tub>Gal80^ts^/+* |
| 2H | “control” as in Fig. 2B, “eIF4A RNAi*”* as in Fig. 2C, “eIF4G1 RNAi*”* as in Fig. 2D, “eIF4E1 RNAi*”* as in Fig. 2E |
| 2I | “control” as in Fig. 2B, “eIF4A RNAi*”* as in Fig. 2C, “eIF4G1 RNAi*”* as in Fig. 2D, “eIF3d1 RNAi*”*: *w/Y; tj-Gal4/+; tub>Gal80^ts^/UAS-eIF3d1 RNAi*  , “eIF2α RNAi”: *w/Y; tj-Gal4/UAS-eIF2α RNAi; tub>Gal80^ts^/+*  “eIF3a RNAi”: *w/Y; tj-Gal4/+; tub>Gal80^ts^/UAS-eIF3a RNAi*, “eIF3h RNAi” as in Fig. 2F, “eIF1A” RNAi as in Fig. 2G |
| 3A | *y,w/Y;; Mi{PT-GFSTF}ImpL2^MI01638-GFSTF^* |
| 3B | As in Fig. 3A |
| 3C | *w/Y; tj-Gal4/+; UAS-upd/+* |
| 3D | As in Fig.3C |
| 3E | *w/Y; tj-Gal4/UAS-eya* |
| 4A | *y,w,hsflp^122^/Y; ubi-GFP, FRT^40A^/FRT^40A^* |
| 4B | *y,w,hsflp^122^/Y; ubi-GFP, FRT^40A^/eIF4A^1013^, FRT^40A^* |
| 4C | *w/Y; tj-Gal4/UAS-LacZ; tub>Gal80^ts^/UAS-LacZ* |
| 4D | *w/Y; tj-Gal4/UAS-LacZ; tub>Gal80^ts^/UAS-upd* |
| 4E | *w/Y; tj-Gal4/UAS-eIF4G1 RNAi; tub>Gal80^ts^/UAS-upd* |
| 4F | “control” as in Fig. 4A, “*eIF4A^1013^*” as in Fig. 4B |
| 4G | “control” as in Fig. 4C, “*LacZ; upd*” as in Fig. 4D, “*eIF4G RNAi; upd*” as in Fig. 4E |
| 5A | *w/Y; tj-Gal4/+; tub>Gal80^ts^/+* |
| 5B | *w/Y; tj-Gal4/+; tub>Gal80^ts^/UAS-eIF3d1 RNAi* |
| 5C | “control” as in Fig. 5A, “eIF3d1 RNAi” as in Fig. 5B |
| 5D | *y,w,hsflp^122^, Tub>Gal4, UAS-nlsGFP/Y;; FRT^82B^ Tub>Gal80/ FRT^82B^ ry^506^* |
| 5E | *y,w,hsflp^122^, Tub>Gal4, UAS-nlsGFP/Y;; FRT^82B^ Tub>Gal80/ FRT^82B^ ry^506^* |
| 5F | “control”: as in Fig.5D&E, “*eIF3d1^EY5735^*”: *y,w,hsflp^122^, Tub>Gal4,UAS-nlsGFP/Y;; FRT^82B^ Tub>Gal80/ FRT^82B^ eIF3d1^EY5735^, “eIF3d1^EP654^”: as in Fig.5G&H* |
| 5G | *y,w,hsflp^122^, Tub>Gal4, UAS-nlsGFP/Y;; FRT^82B^ Tub>Gal80/ FRT^82B^ eIF3d1^EP654^* |
| 5H | *y,w,hsflp^122^, Tub>Gal4, UAS-nlsGFP/Y;; FRT^82B^ Tub>Gal80/ FRT^82B^ eIF3d1^EP654^* |
| 5I | As in Fig.5F |
| 6A | *w/Y; tj-Gal4/+; tub>Gal80^ts^/+* |
| 6B | *w/Y; tj-Gal4/UAS-CkIIα RNAi (2); tub>Gal80^ts^/+* |
| 6C | *w/Y; tj-Gal4/+; tub>Gal80^ts^/UAS-CkIIβ RNAi (1)* |
| 6D | *“*control*, CkIIα RNAi (2), CkIIβ RNAi (1)”:* as in Fig. 6A-C*,* “*CkIIα RNAi (1)*”: *w/Y; tj-Gal4/+; tub>Gal80^ts^/UAS-CkIIα RNAi (1),* “*CkIIβ RNAi (2)*”: *w/Y; tj-Gal4/+; tub>Gal80^ts^/UAS-CkIIβ RNAi (2).* |
| 6E | As in Fig. 6D |
| 6F | *“*control”: *w/Y; tj-Gal4/UAS-LacZ; tub>Gal80^ts^/UAS-LacZ,* “*LacZ, CkIIα RNAi(1)*”*: w/Y; tj-Gal4/UAS-LacZ; tub>Gal80^ts^/UAS-CkIIα RNAi (1),* “*eIF3d1^DD^, CkIIα RNAi(1)*”: *w/Y; tj-Gal4/UAS-eIF3d1^DD^; tub>Gal80^ts^/UAS-CkIIα RNAi (1),* “*eIF3d1^NN^, CkIIα RNAi(1)*”: *w/Y; tj-Gal4/UAS-eIF3d1^NN^; tub>Gal80^ts^/UAS-CkIIα RNAi (1),* “*eIF3d1^WT^, CkIIα RNAi(1)*”: *w/Y; tj-Gal4/UAS-eIF3d1^WT^; tub>Gal80^ts^/UAS-CkIIα RNAi (1).* |
| 7A | *w/Y; tj-Gal4/UAS-LacZ; tub>Gal80^ts^/UAS-upd* |
| 7B | *w/Y; tj-Gal4/UAS-CkIIα RNAi (1); tub>Gal80^ts^/UAS-upd* |
| 7C | As in Fig. 7A,B. |
| 7D | *w/Y; tj-Gal4, tub>Gal80^ts^/UAS-LacZ; FRT^82B^ Stat92E^397^, ca, e/ mwh red Stat92E^Frankenstein^ e* |
| 7E | *w/Y; tj-Gal4, tub>Gal80^ts^/UAS-CkIIα; FRT^82B^ Stat92E^397^, ca, e/ mwh red Stat92E^Frankenstein^ e* |
| 7F | As in Fig. 7D&E |
| 7G | As in Fig. 7D |
| 7H | As in Fig. 7E |
| 7I | As in Fig. 7D&E |
| 8A | *yw/Y;; Mi{PT-GFSTF}ImpL2^MI01638-GFSTF^* |
| 8C | *w/Y;; FRT^82B^ Stat92E^397^, ca, e/ +* |
| 8D | *w/Y;; FRT^82B^ Stat92E^397^, ca, e/ mwh red Stat92E^Frankenstein^ e* |
| 8E | As in Fig. 8C&D |
| S1A,B | *w/Y; tj-Gal4/ UAS-Rbf RNAi; tub>Gal80^ts^/+* |
| S2A | *y,w,hsflp^122^ ,Tub>Gal4, UAS-nlsGFP/Y; Tub>Gal80, FRT^40A^/FRT^40A^* |
| S2B | As in Fig. S2A |
| S2C | *y,w,hsflp^122^ ,Tub>Gal4, UAS-nlsGFP/Y; Tub>Gal80, FRT^40A^/eIF4A^1013^, FRT^40A^* |
| S2D | As in Fig. S2C |
| S2E | “control” as in Fig. S2A, “*eIF4A^1013^*” as in Fig. S2B, “*eIF4A^1006^*”: *y,w,hsflp^122^ ,Tub>Gal4, UAS-nlsGFP/Y; Tub>Gal80, FRT^40A^/eIF4A^1006^, FRT^40A^* |
| S2F | As in Fig. S2E |
| S2G | “control”: *y,w,hsflp^122^/Y; ubi-GFP, FRT^40A^/FRT^40A^*_,_ “*eIF4A^1013^*”: *y,w,hsflp^122^/Y; ubi-GFP, FRT^40A^/eIF4A^1013^, FRT^40A^* |
| S2H | As in Fig. S2G |
| S2I | *y,w,hsflp^122^/Y; ubi-GFP, FRT^40A^/FRT^40A^* |
| S2J | *y,w,hsflp^122^/Y; ubi-GFP, FRT^40A^/eIF4A^1013^, FRT^40A^* |
| S2K | *y,w,hsflp^122^/Y;; ubi-GFP, FRT^80B^/FRT^80B^* |
| S2L | *y,w,hsflp^122^/Y;; ubi-GFP, FRT^80B^/eIF4E1^S058911^, FRT^80B^* |
| S2M | “control” as in Fig. S2K, “*eIF4E1^S058911^”* as in Fig. S2L |
| S2N | As in Fig. S2M |
| S3A | *w/Y; tj-Gal4/+; tub>Gal80^ts^/+* |
| S3B | *w/Y; tj-Gal4/UAS-eIF4G1 RNAi; tub>Gal80^ts^/+* |
| S3C | *w/Y; tj-Gal4/UAS-LacZ; tub>Gal80^ts^/UAS-LacZ* |
| S3D | *w/Y; tj-Gal4/UAS-LacZ; tub>Gal80^ts^/UAS-P35* |
| S3E | “control” as in Fig. S3D, “LacZ; P35” as in Fig. S3E |
| S3F | “*LacZ; LacZ*”: *w/Y; tj-Gal4/UAS-LacZ; tub>Gal80^ts^/UAS-LacZ,* “*LacZ, P35*”: *w/Y; tj-Gal4/UAS-LacZ; tub>Gal80^ts^/UAS-P35,* “*P35, LacZ*”: *w/Y; tj-Gal4/UAS-P35; tub>Gal80^ts^/UAS-LacZ,* "*eIF4G1 RNAi, LacZ*”: *w/Y; tj-Gal4/UAS-eIF4G1 RNAi; tub>Gal80^ts^/UAS-LacZ, “eIF4G1 RNAi, P35”*: *w/Y; tj-Gal4/UAS-eIF4G1 RNAi; tub>Gal80^ts^/UAS-P35,* “*LacZ; eIF4A RNAi*”: *w/Y; tj-Gal4/UAS-LacZ; tub>Gal80^ts^/UAS-eIF4A RNAi,* “P35*; eIF4A RNAi*”: *w/Y; tj-Gal4/UAS-P35; tub>Gal80^ts^/UAS-eIF4A RNAi.* |
| S4A | *y,w,hsflp^122^/Y; Tub>Gal80, FRT^40A^/FRT^40A^; Tub>Gal4, UAS-LifeAct-mCherry/+* |
| S4B,C | *y,w,hsflp^122^/Y; Tub>Gal80, FRT^40A^/eIF4A^1013^, FRT^40A^; Tub>Gal4, UAS-LifeAct-mCherry/+* |
| S5A | *w/Y; tj-Gal4/+; tub>Gal80^ts^/+* |
| S5B | *w/Y; tj-Gal4/UAS-eIF2α RNAi; tub>Gal80^ts^/+* |
| S5C | *w/Y; tj-Gal4/+; tub>Gal80^ts^/UAS-eIF3a RNAi*, |
| S5D | As in Fig. S5A-C, 2F&G |
| S6A | *w/Y; tj-Gal4/+; tub>Gal80^ts^/+* |
| S6B | *w/Y; tj-Gal4/UAS-eIF4A RNAi; tub>Gal80^ts^/+* |
| S6D | *w/Y; tj-Gal4/+; tub>Gal80^ts^/UAS-eIF4A RNAi* |
| S6E | “control” as in Fig. 4C, “*LacZ; upd*” as in Fig. 4D, “*eIF4A RNAi; upd*” as in Fig. S6D, “*eIF3d1 RNAi, upd*”; *w/Y; tj-Gal4/+; tub>Gal80^ts^/UAS-eIF3d1 RNAi, UAS-upd.* |
| S6F | *w/Y; tj-Gal4/+; tub>Gal80^ts^/UAS-eIF3d1 RNAi* |
| S6G | “*LacZ; LacZ*”: *w/Y; tj-Gal4/UAS-LacZ; tub>Gal80^ts^/UAS-LacZ,* “*P35, LacZ*”: *w/Y; tj-Gal4/UAS-P35; tub>Gal80^ts^/UAS-LacZ,* "LacZ, *eIF3d1 RNAi*”: *w/Y; tj-Gal4/UAS-LacZ; tub>Gal80^ts^/UAS-eIF3d1 RNAi,* "P35, *eIF3d1 RNAi*”: *w/Y; tj-Gal4/UAS-P35; tub>Gal80^ts^/UAS-eIF3d1 RNAi.* |
| S6H | *w/Y; tj-Gal4/+; tub>Gal80^ts^/UAS-eIF3d1 RNAi* |
| S6I | *w/Y; tj-Gal4/+; tub>Gal80^ts^/UAS-eIF3d1 RNAi, UAS-upd* |
| S7B | *“*control”: *w/Y; tj-Gal4/+; tub>Gal80^ts^/+,* “*eIF3d1^WT^*”*: w/Y; tj-Gal4/UAS-eIF3d1^WT^; tub>Gal80^ts^/+,* “*eIF3d1^DD^*”: *w/Y; tj-Gal4/UAS-eIF3d1^DD^; tub>Gal80^ts^/+,* “*eIF3d1^NN^*”: *w/Y; tj-Gal4/UAS-eIF3d1^NN^; tub>Gal80^ts^/+.* |
| S7C | *“*control”: *w/Y; tj-Gal4/+,* “*eIF3d1^DD^*”: *w/Y; tj-Gal4/UAS-eIF3d1^DD^,* “*eIF3d1^NN^*”: *w/Y; tj-Gal4/UAS-eIF3d1^NN^.* |
| S7D | *“*control”: *w/Y; tj-Gal4/+; tub>Gal80^ts^/+,* “*eIF3d1^WT^*”*: w/Y; tj-Gal4/+; tub>Gal80^ts^/ UAS-eIF3d1^WT^,* “*eIF3d1^helix11^*”: *w/Y; tj-Gal4/+; tub>Gal80^ts^/ UAS-eIF3d1^helix11^*. |
| S8A | *w/Y; tj-Gal4/+; tub>Gal80^ts^/+* |
| S8B | *w/Y; tj-Gal4/+; tub>Gal80^ts^/UAS-CkIIβ RNAi (2)* |
| S8C | As in Fig. S8A-B |
| S8D | *w/Y; tj-Gal4/+; tub>Gal80^ts^/UAS-CkIIα RNAi (2)* |
| S8E | “control”: as in Fig. S6A, “*CkIIα RNAi (2)*”: as in Fig S8D. |
