# Supplementary figures and images for: Signals from the niche promote distinct modes of translation initiation to control stem cell differentiation and renewal in the Drosophila testis
Source: PLoS Biol. 2025 Mar 11;23(3):e3003049. doi: 10.1371/journal.pbio.3003049 (PMC12136000; doi:10.1371/journal.pbio.3003049)

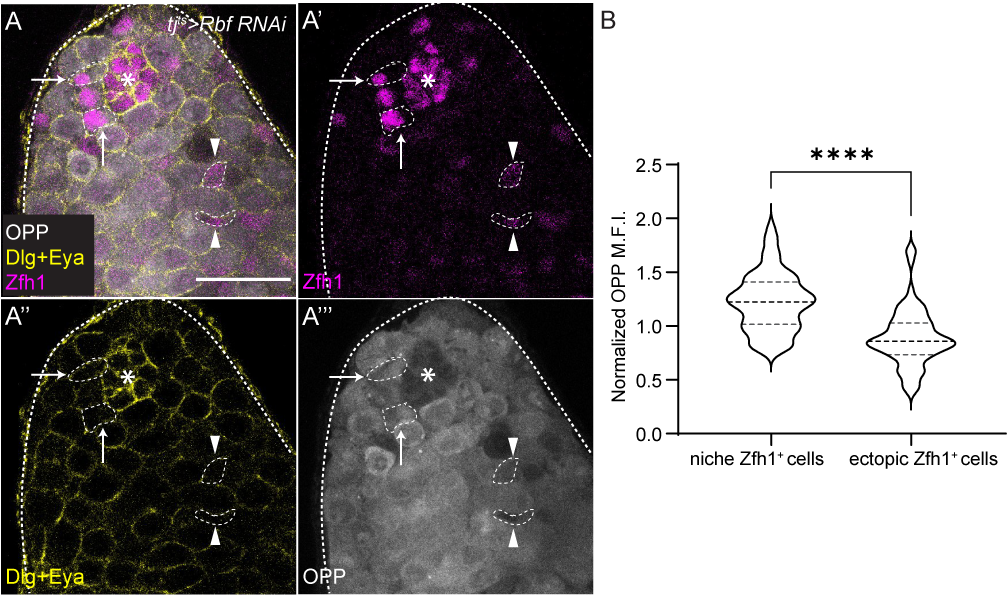

Supplement: S1 Fig — (A) A testis in which Rbf was knocked down by RNAi, resulting in ectopic CySCs away from the hub and absence of differentiated cyst cells (marked with Eya, yellow). OPP incorporation (white) shows that the global translation rate in CySCs adjacent to the hub (arrows) is higher than in ectopic CySCs (arrowheads). Zfh1 (magenta) labels CySCs and Dlg (yellow) labels cell outlines. Asterisks indicate the hub. Scale bar: 15 µM. (B) Quantification of OPP incorporation in niche CySCs and ectopic CySCs, normalized to the hub (N ≥ 67 cells from 9 testes, Student t test, ****P < 0.0001). Underlying data for all graphs can be found in file S1 Data. (TIF) [file pbio.3003049.s004.tif]

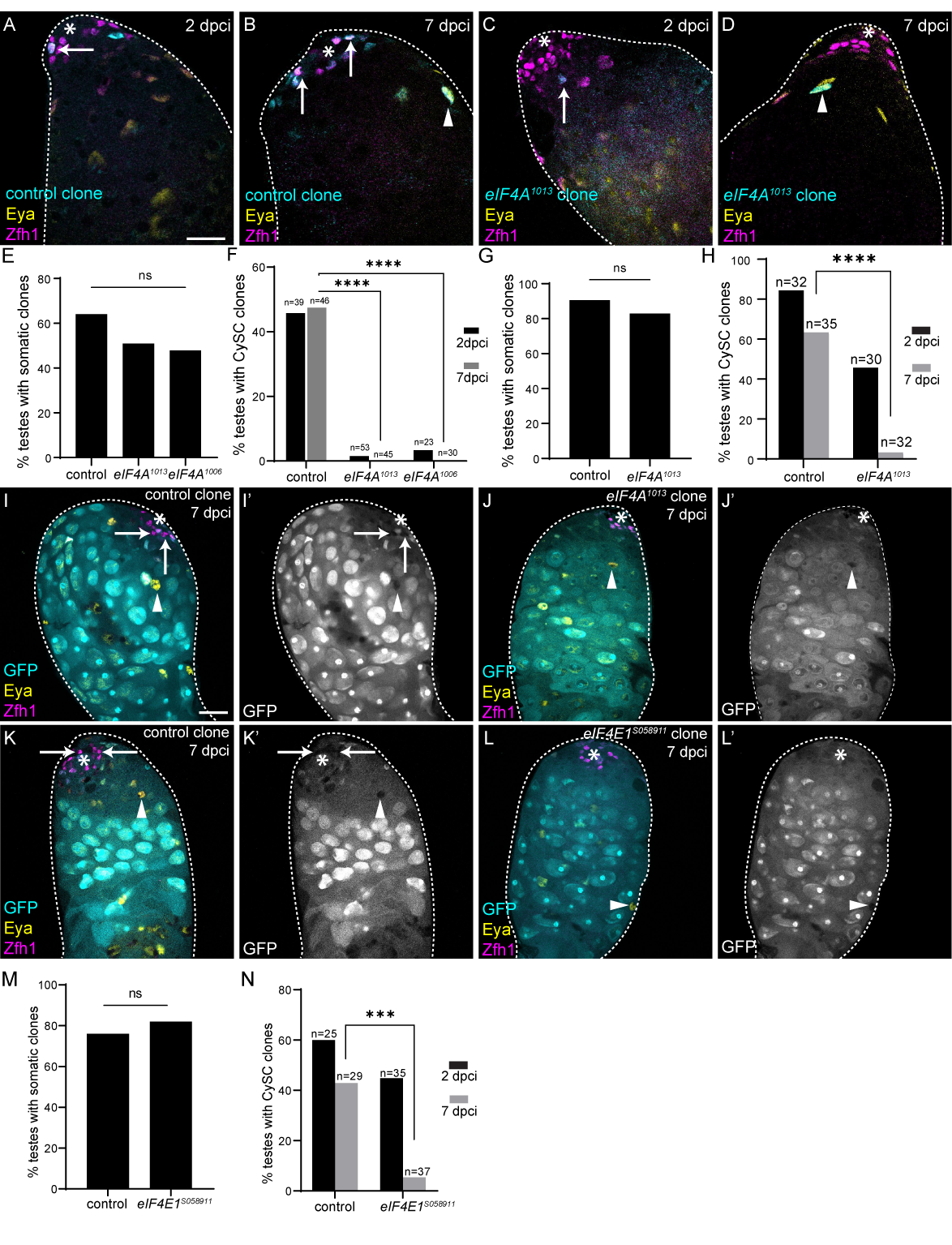

Supplement: S2 Fig — (A–D) Testes with positively marked control clones (A,B) or clones homozygous for eIF4A1013 (C,D) at 2 dpci (A,C) and 7 dpci (B,D). GFP (cyan) labels the clone. Control clones are readily recovered in CySCs at 2 dpci and maintained at 7 dpci, while mutant clones are rarely observed adjacent to the hub. Zfh1 (magenta) labels CySCs and Eya (yellow) labels differentiated cells. Arrows mark CySCs and arrowheads mark differentiated cells. Asterisks indicate the hub. Scale bar: 15 µM. (E) Percentage of testes with positively-marked control or eIF4A mutant clones in either CySCs or differentiated cyst cells at2 dpci (N ≥ 27 testes, Chi-squared test, ns P = 0.3443). (F) Percentage of testes with positively-marked control or eIF4A mutant CySC clones at 2 dpci and 7 dpci. (N ≥ 27 testes, Chi-squared test, **** P < 0.0001). (G) Percentage of testes with negatively-marked control or eIF4A mutant clones in either CySCs or differentiated cyst cells at 2 dpci (N ≥ 30 testes, Chi-squared test, ns P = 0.3517). (H) Percentage of testes with negatively-marked control or eIF4A mutant CySC clones at 2 dpci and 7 dpci. (N ≥ 30 testes, Chi-squared test, **** P < 0.0001). (I–J′) Testes with negatively-marked control clones (E, E′) and clones homozygous mutant for eIF4A1013 (F, F′) at 7 dpci. Clones are identified by lack of GFP (cyan). Mutant CySC clones are not recovered. Zfh1 (magenta) labels CySCs and Eya (yellow) labels differentiated cells. Arrows mark CySCs and arrowheads mark differentiated cells. Asterisks indicate the hub. Scale bar: 15 µM. (K–L′) Testes with negatively-marked control clones (G, G′) and clones homozygous mutant for eIF4E1S058911 (H, H′) at 7 dpci. Clones are identified by lack of GFP (cyan). Mutant CySC clones are not recovered. Zfh1 (magenta) labels CySCs and Eya (yellow) labels differentiated cells. Arrows mark CySCs and arrowheads mark differentiated cells. Asterisks indicate the hub. Scale bar: 15 µM. (M) Percentage of testes with negatively-marked control or [file pbio.3003049.s005.tif]

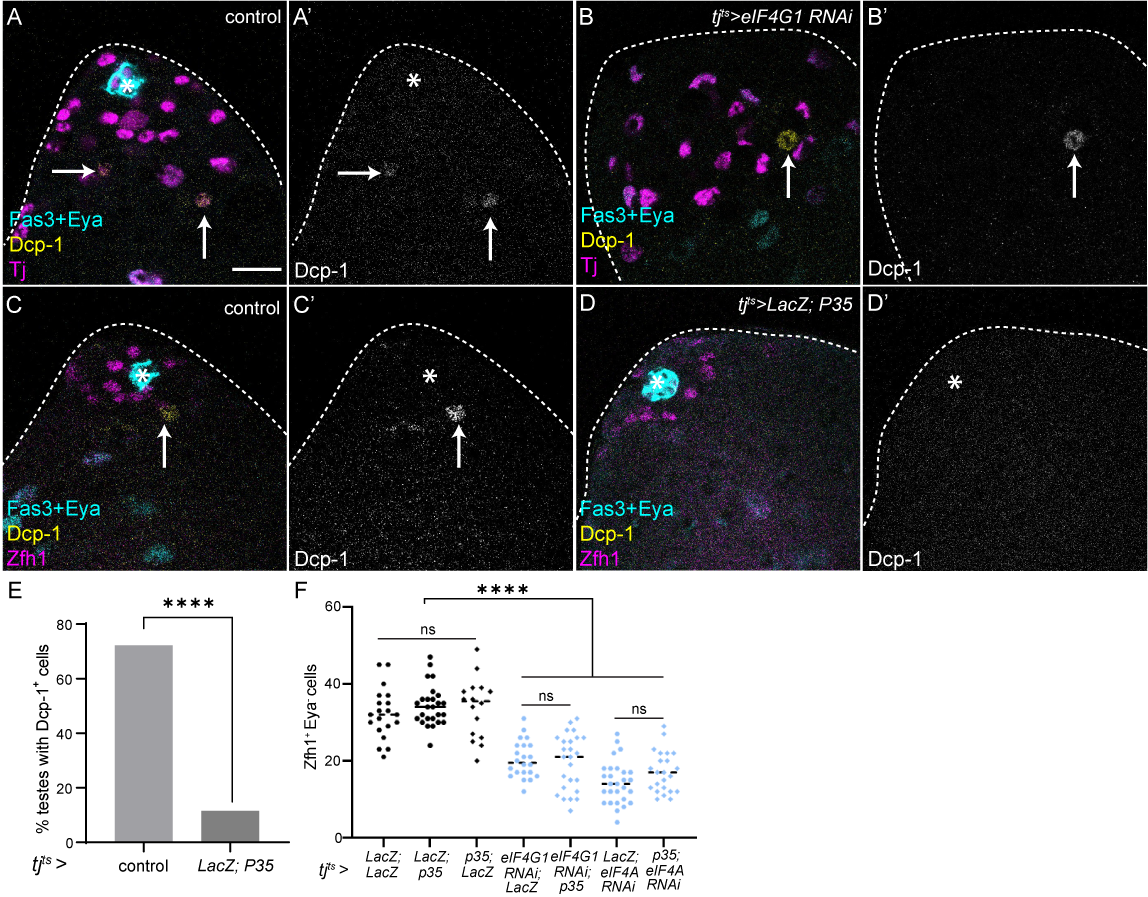

Supplement: S3 Fig — (A, B) A control (tjts>+) testis (A) and a testis in which eIF4G1 was knocked down for 7 days (B), labeled with antibodies against the activated caspase, Dcp-1 (yellow, single channel A′,B′) to mark apoptotic cells, Tj (magenta) to label CySCs and early cyst cells, and Eya and Fas3 (cyan) to label late-stage cyst cells and the hub respectively. No increase in Dcp-1-positive cells (arrows) is visible. Scale bar: 15 µM. (C, D) Dcp-1 expression in control (tjts>LacZ; LacZ) testis (C) and a testis in which the baculovirus caspase inhibitor was over-expressed in cyst cells (tjts>LacZ; P35) (D). Dcp-1 positive cells (arrows) are rarely observed in testes with P35 over-expression compared to the control. Tj (magenta) labels early cyst cells, Eya and Fas3 (cyan) labels late stage cyst cells and the hub respectively, and Dcp-1 (yellow) labels cells undergoing apoptosis. Scale bar: 15 µM. (E) Percentage of testes with Dcp-1-positive cells in control and testes in which P35 was over-expressed in the cyst lineage (N ≥ 18 testes, Chi-squared test, **** P < 0.0001). (F) Number of Zfh1+ Eya− CySCs in testes in which the indicated initiation factors were knocked down together with inhibition of apoptosis by co-expression of P35. Blocking cell death did not rescue CySC loss caused by knockdown of initiation factors (N ≥ 15 testes, Kruskal–Wallis test, ****P < 0.0001, ns P > 0.9999). Underlying data for all graphs can be found in file S1 Data. (TIF) [file pbio.3003049.s006.tif]

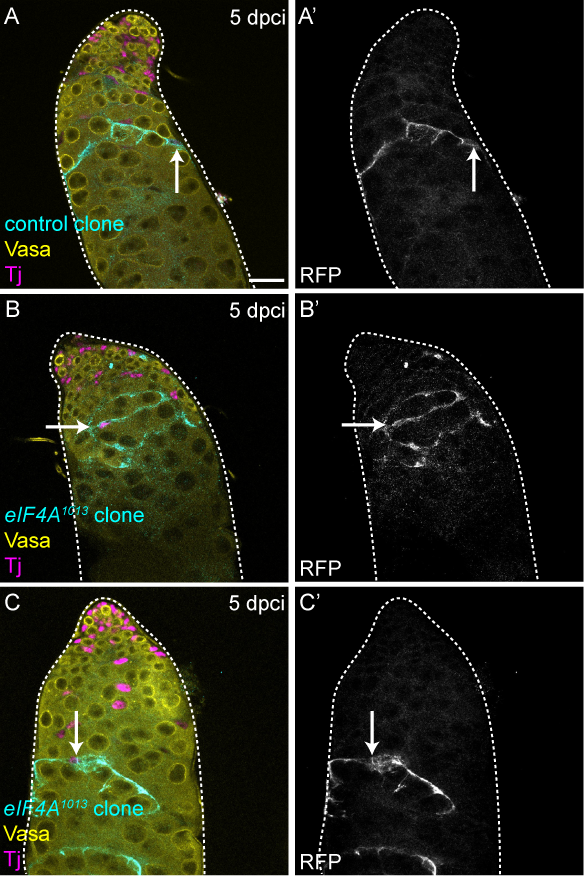

Supplement: S4 Fig — (A–C) Testes with membrane-labeled control clones (A, A′) and two examples of clones homozygous mutant for eIF4A1013 (B–C′) at 5 dpci. RFP (cyan) labels the clones (arrows). Both control and mutant clones display the characteristic morphology of cyst cells, with a long flattened cytoplasm enveloping germ cell cysts (arrows). Tj (magenta) labels cyst cells and Vasa (yellow) labels germ cells. Scale bar: 15 µM. (TIF) [file pbio.3003049.s007.tif]

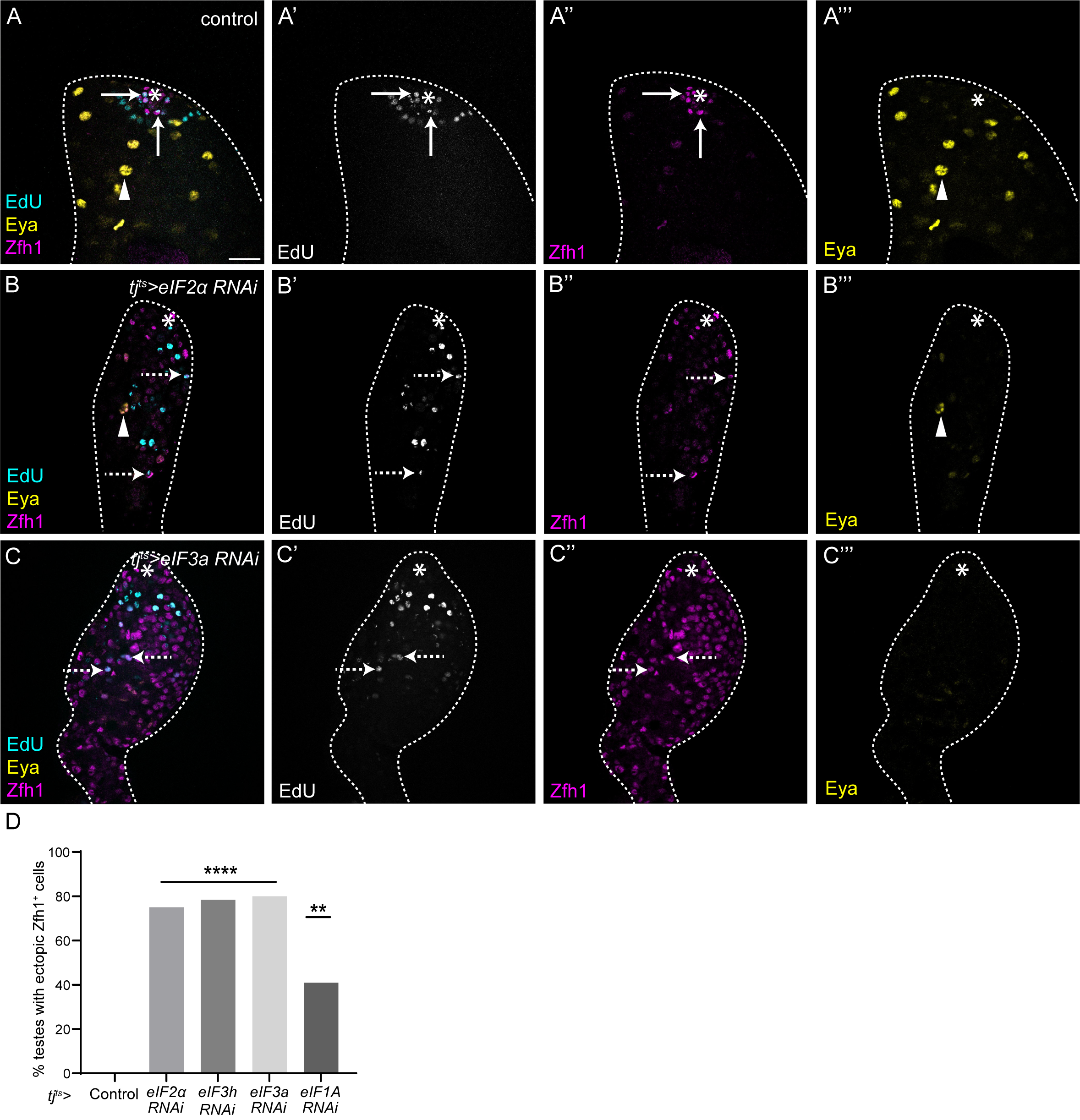

Supplement: S5 Fig — (A–C‴) A control (tjts> +) testis (A–A‴), and testes in which eIF2α (B–B‴) or eIF3a was knocked down in CySCs (C–C‴). EdU (cyan) labels cells in S phase. In controls, EdU-positive somatic cells are found only adjacent to the hub, while in eIF knockdowns, EdU-positive CySC-like cells are observed away from the hub. Zfh1 (magenta) labels CySCs and Eya (yellow) labels differentiated cyst cells. Arrows mark CySCs, dashed arrows indicate ectopic Zfh1-positive cells away from the hub, and arrowheads mark differentiated cells. Asterisks indicate the hub. Scale bar: 15 µM. (D) Percentage of testes with Edu+ Zfh1+ cells at least 2 cell diameters from the hub (N ≥ 15 testes, Chi-squared test, ****P < 0.0001, **P < 0.01). Underlying data for all graphs can be found in file S1 Data. (TIF) [file pbio.3003049.s008.tif]

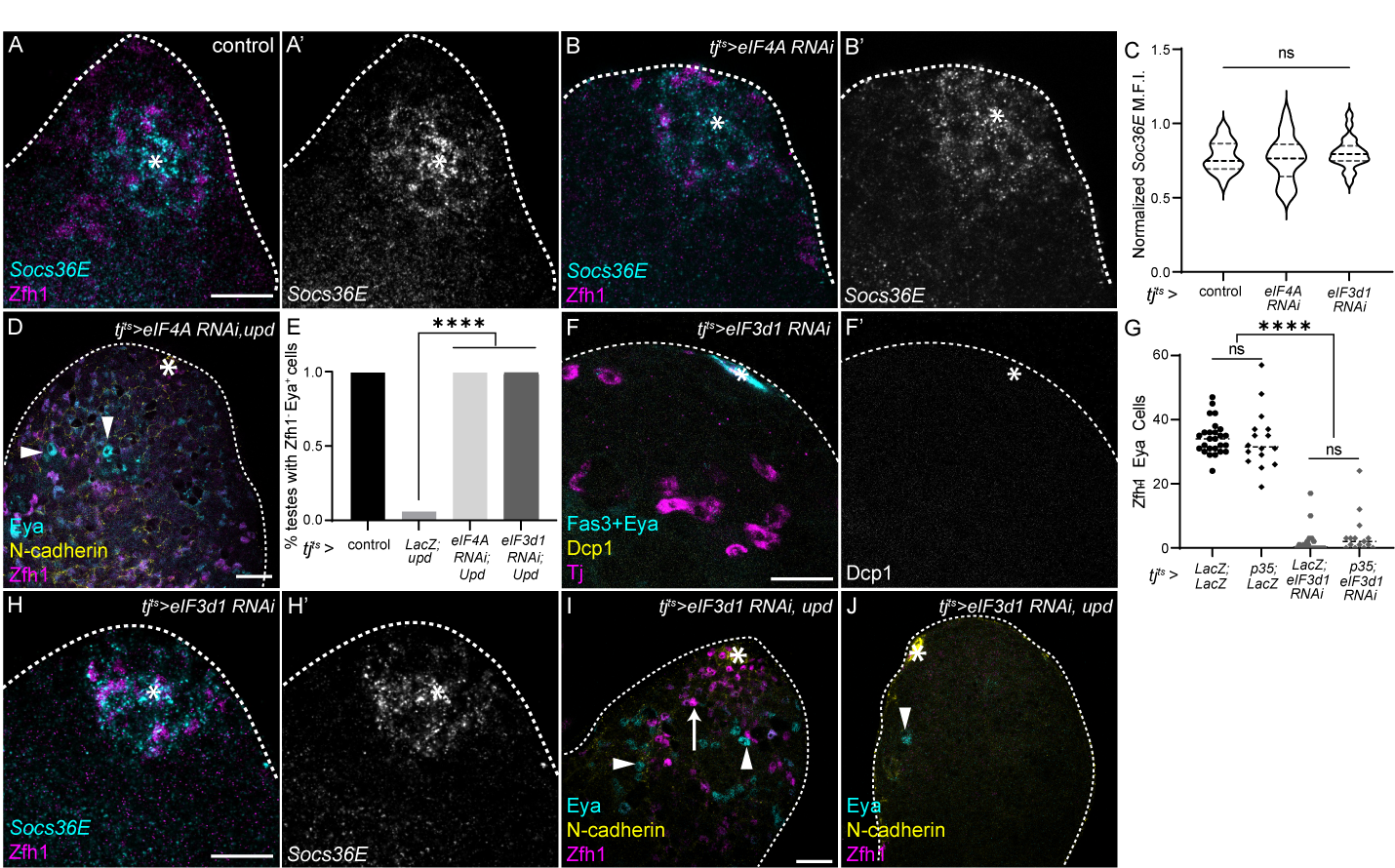

Supplement: S6 Fig — (A, B) Expression of the JAK/STAT target Socs36E detected by in situ hybridization chain reaction (cyan, single channel in A′,B′) in a control (tjts> +) testis (A), and a testis in which eIF4A (B) was knocked down in CySCs, after 2 days at 29 °C. No apparent change in Soc36E mRNAs is visible. Zfh1 (magenta) labels CySCs. Asterisks indicate the hub. Scale bar: 15 µM. (C) Quantification of Socs36E fluorescence signal in CySCs around the hub in the indicated genotypes, normalized to the levels in the hub (N ≥ 20 testes, Šidák multiple comparisons test, ns P > 0.9). (D) A testis in which Upd was over-expressed with concomitant knockdown of eIF4A in CySCs (tjts > eIF4A RNAi, upd). N-cadherin (yellow) labels the hub. Zfh1 (magenta) labels CySCs and Eya (cyan) labels differentiated cyst cells. Arrows mark CySCs and arrowheads mark differentiated cells. Asterisks indicate the hub. Scale bar: 15 µM. (E) Percentage of testes with Eya-positive, Zfh1-negative differentiating cyst cells in the indicated genotypes (N ≥ 15 testes, Chi-squared test, ****P < 0.0001). (F) Detection of cell death in a testis in which eIF3d1 was knocked down in CySCs for 7 days with an antibody against activated Dcp-1 (yellow). Dcp-1-positive cells are rarely observed. Tj (magenta) labels early cyst cells, Eya and Fas3 (cyan) label cyst cells and the hub respectively. Scale bar: 15 µM. (G) Quantification of the number of Zfh1+ Eya− CySCs in control or eIF3d1 knockdown testes, with or without inhibition of apoptosis using the baculovirus caspase inhibitor P35. Blocking cell death did not rescue CySC numbers upon eIF3d1 knockdown (N ≥ 15 testes, Kruskal–Wallis test, ****P < 0.0001, ns P > 0.9999). (H) Expression of the JAK/STAT target Socs36E detected by in situ hybridization chain reaction (cyan, single channel in H′) in a testis in which eIF3d1 was knocked down in CySCs, after 2 days at 29 °C. Zfh1 (magenta) labels CySCs. Asterisks indicate the hub. Scale bar: 15 µM. (I–J) Testes in which Upd was over- [file pbio.3003049.s009.tif]

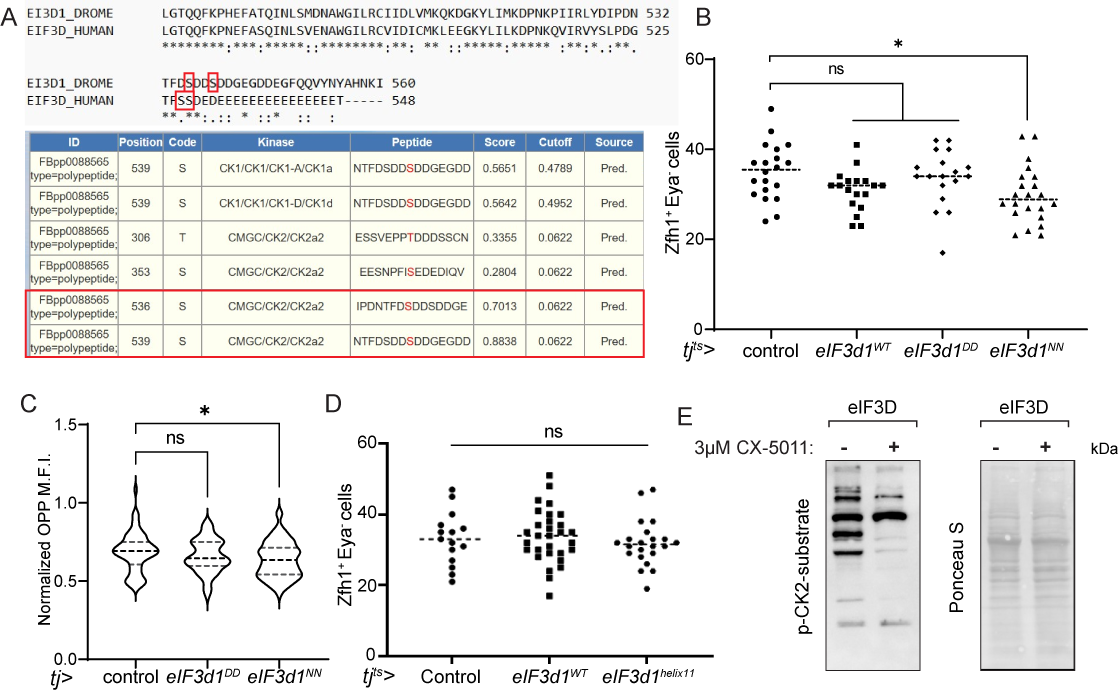

Supplement: S7 Fig — (A) Top: alignment of the N terminus of human eIF3d and Drosophila eIF3d1. Asterisks indicate conserved residues, colons indicate residues with strongly similar properties and periods indicate residues with weakly similar properties. The two serines subject to phosphorylation in human eIF3d are highlighted in red boxes along with predicted phosphorylation sites in Drosophila. Bottom: predicted phosphorylation sites in Drosophila eIF3d1 using Group-based Prediction System (GPS) server. The boxed lines indicate serines 536 and 539, highlighted above, which are strong predicted targets of CkII. (B) Number of Zfh1+ Eya− CySCs in testes in which wild-type (eIF3d1WT), phospho-mimetic (eIF3d1DD) or phospho-dead (eIF3d1NN) eIF3d1 was over-expressed. Phospho-dead eIF3d1 expression results in a significant decrease in CySC numbers (N ≥ 18 testes, * P < 0.05, ns P = 0.1150 (eIF3d1WT) or P > 0.9999 (eIF3d1DD), Kruskal-Wallis test). (C) Quantification of OPP levels in CySCs in indicated genotypes, normalized to the GSCs (N ≥ 62 cells from ≥ 8 testes, Šidák multiple comparisons test, ns P = 0.275, *P = 0.023). (D) Number of Zfh1+ Eya− CySCs in testes in which wild-type eIF3d1 or a form of eIF3d1 in which mutations are introduced in the mRNA 5′ m7G cap-binding domain (eIF3d1helix11) were over-expressed (N ≥ 15 testes, Kruskal–Wallis test, ns P > 0.9999). (E) Western blot from lysates of cells expressing a control construct (RAP2A) or eIF3d and labeled with an antibody against a pan-phospho-CK2 substrate (left) or stained for total protein (right). Addition of the CK2 inhibitor CX-5011 resulted in decreased labeling for the phospho-CK2 substrate. Underlying data for all graphs can be found in file S1 Data, raw images of western blots can be found in file S1 Raw Images. (TIF) [file pbio.3003049.s010.tif]

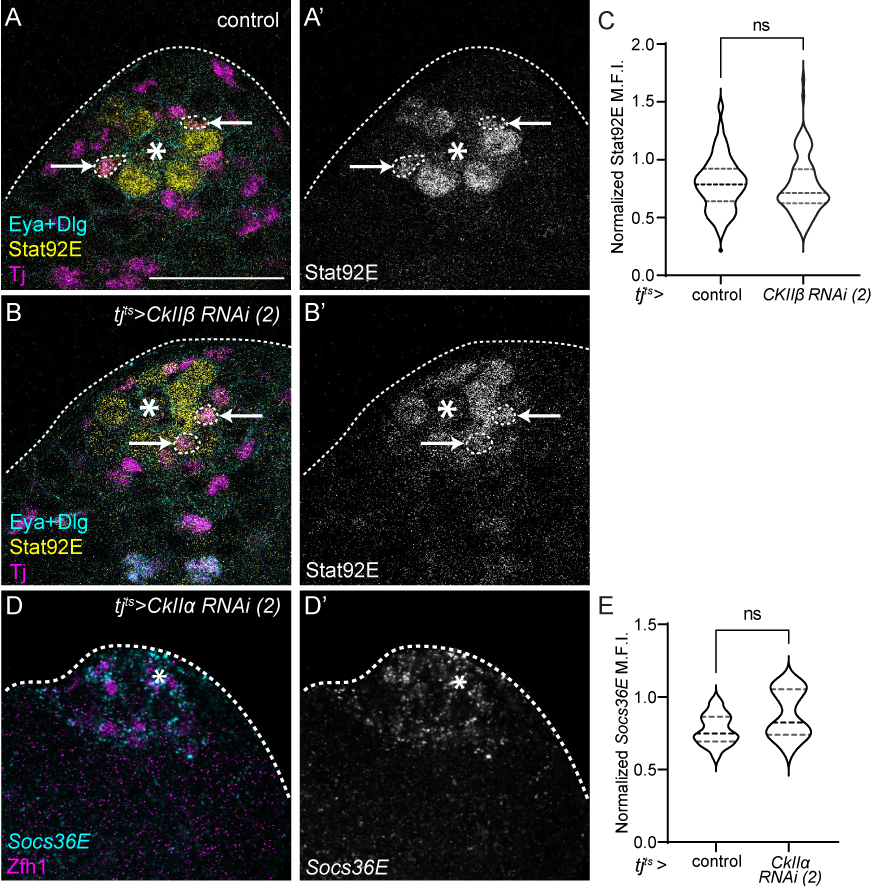

Supplement: S8 Fig — (A, B) A control testis (A) and a testis in which CkIIβ was knocked down in CySCs (B), stained with an antibody against Stat92E (yellow, single channel A′,B′) after 20h at the restrictive temperature. Tj (magenta) labels CySCs and early cyst cells, Dlg (cyan) labels the hub and Eya (cyan) labels differentiated cyst cells. Arrows mark CySCs. Asterisks indicate the hub. Scale bar: 15 µM. (C) Quantification of Stat92E levels in CySCs in control testes or upon CkIIβ knockdown, normalized to levels in neighboring GSCs. (N > 100 cells from ≥ 7 testes, Student t test, ns P = 0.5057) (D) Expression of the JAK/STAT target Socs36E detected by in situ hybridization chain reaction (cyan, single channel in D′) in a testis in which CkIIα was knocked down in CySCs for 2 days. Zfh1 (magenta) labels CySCs. Asterisks indicate the hub. Scale bar: 15 µM. (E) Quantification of Socs36E fluorescence signal in CySCs around the hub, normalized to the levels in the hub (N ≥ 16 testes, Šidák multiple comparisons test, ns P = 0.06). Underlying data for all graphs can be found in file S1 Data. (TIF) [file pbio.3003049.s011.tif]
