# Supplementary material for: Signals from the niche promote distinct modes of translation initiation to control stem cell differentiation and renewal in the Drosophila testis
Source: PLoS Biol. 2025 Mar 11;23(3):e3003049. doi: 10.1371/journal.pbio.3003049 (PMC12136000; doi:10.1371/journal.pbio.3003049)
Supplement: S1 Raw Images — (PDF) [file pbio.3003049.s013.pdf]

CORRESPONDING TO FIG 6G

INPUT SAMPLES: eIF3d WT

primary -> antigene specific
secondary -> rabbit

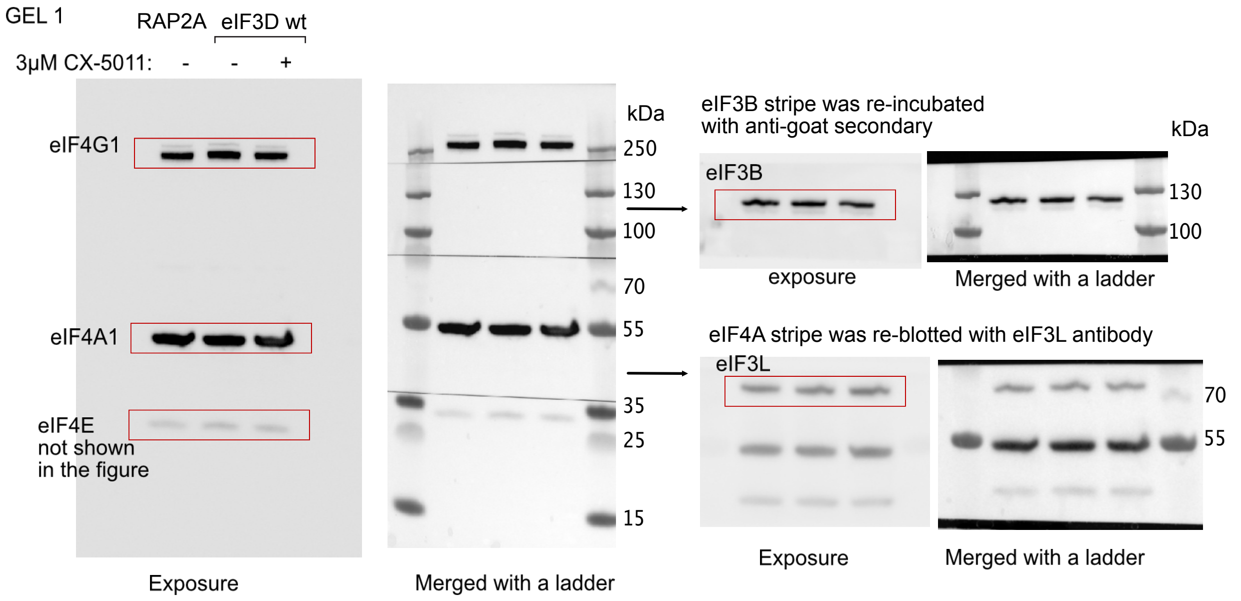

GEL 2 (loaded the same sample as at GEL1)

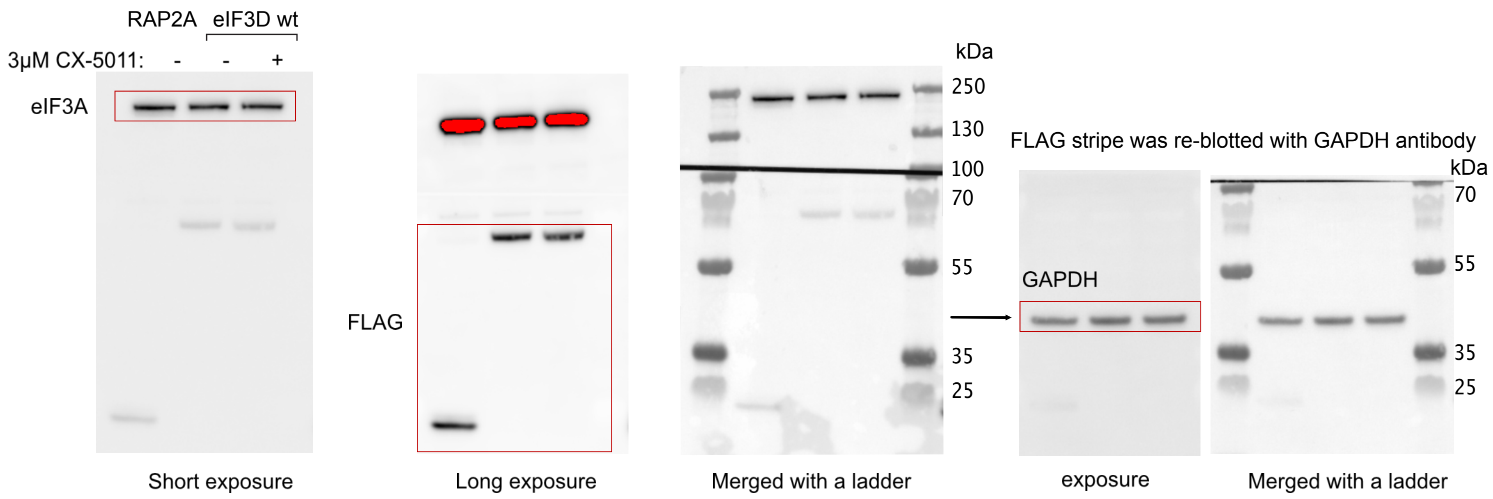

IP SAMPLES: eIF3D WT

primary -> antigene specific
secondary -> rabbit

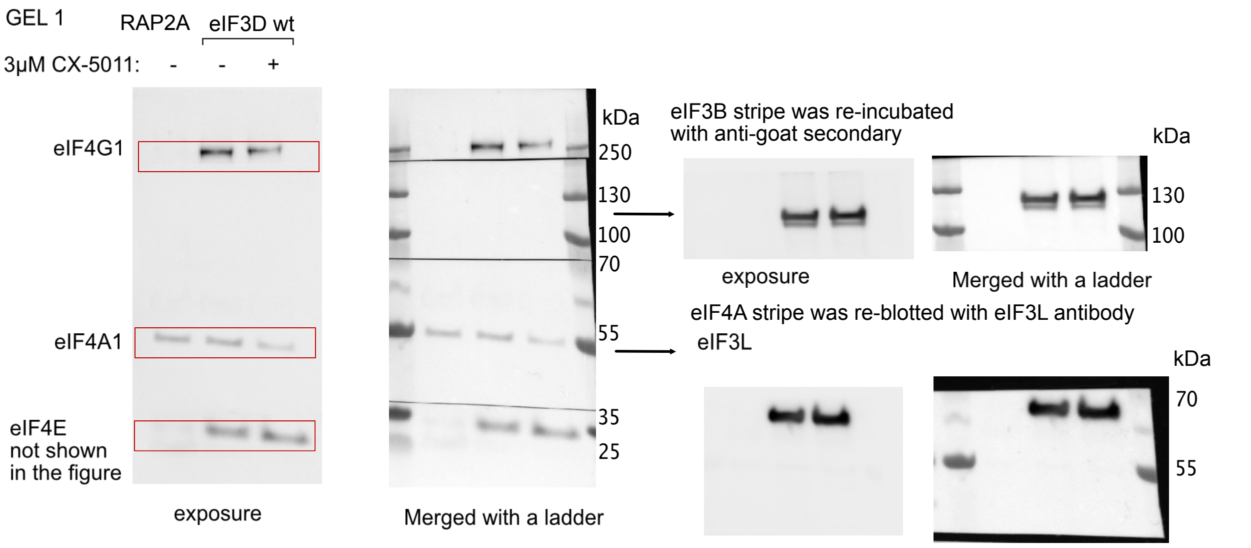

GEL 2 (loaded the same sample as at GEL1)

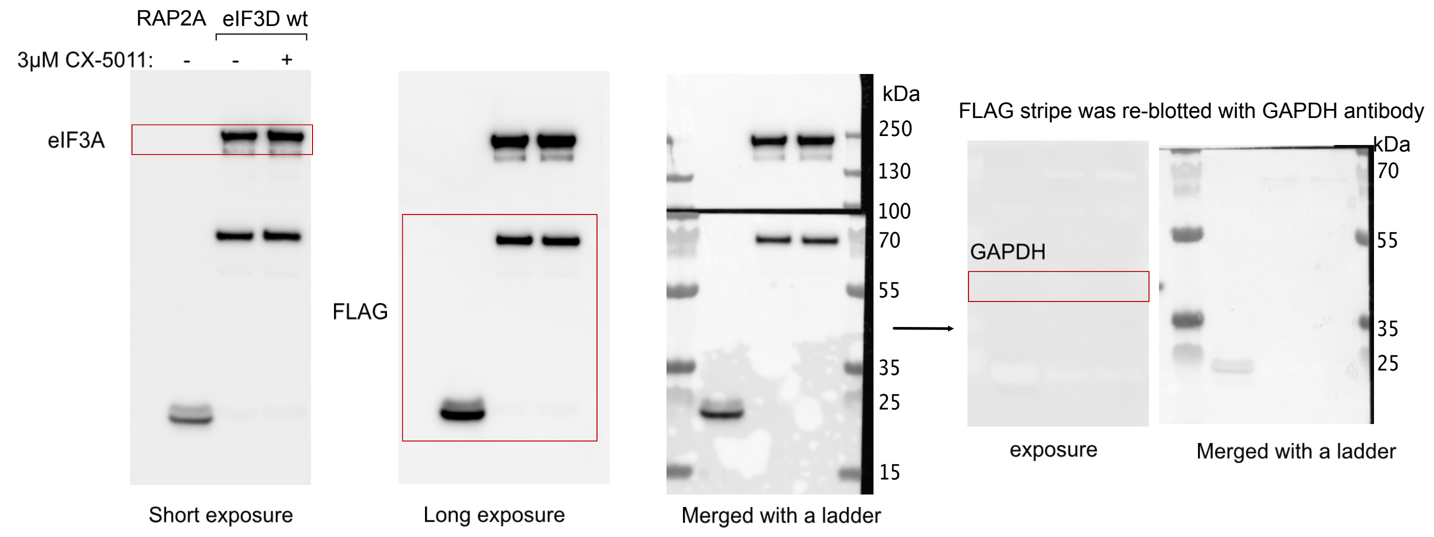

CORRESPONDING TO FIG 6H

INPUT SAMPLES: eIF3D DD

primary -> antigene specific
secondary -> eIF3B goat all other antibodies rabbit

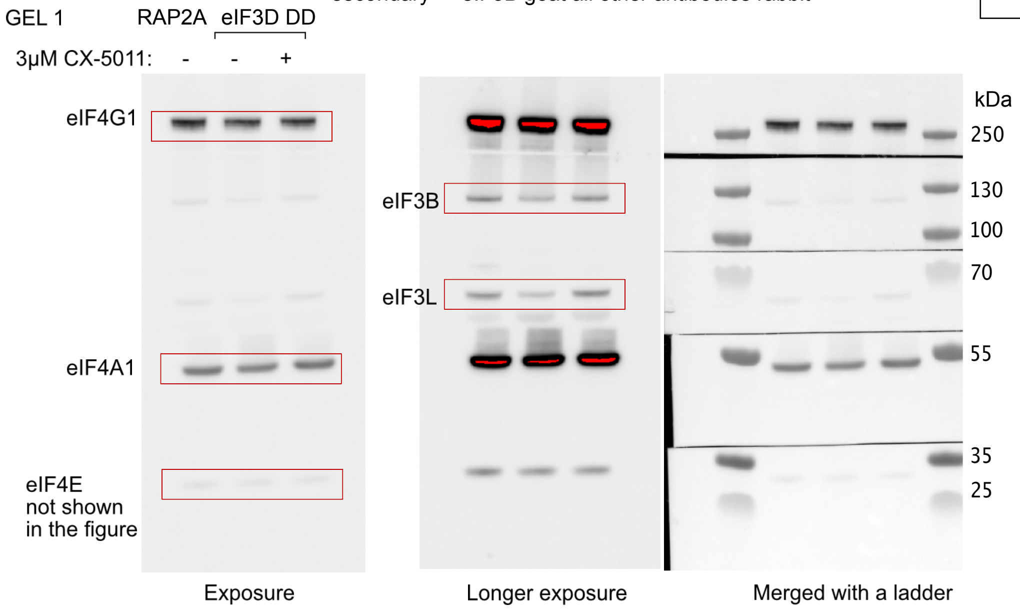

GEL 2 (loaded the same sample as at GEL1)

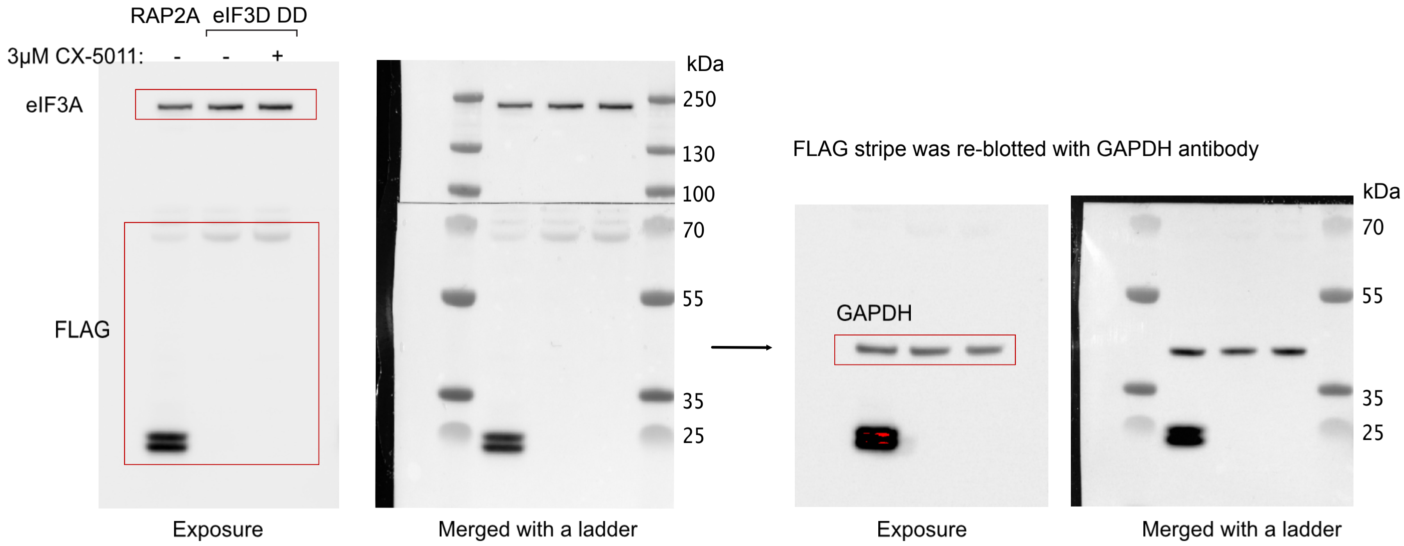

IP SAMPLES: eIF3D DD

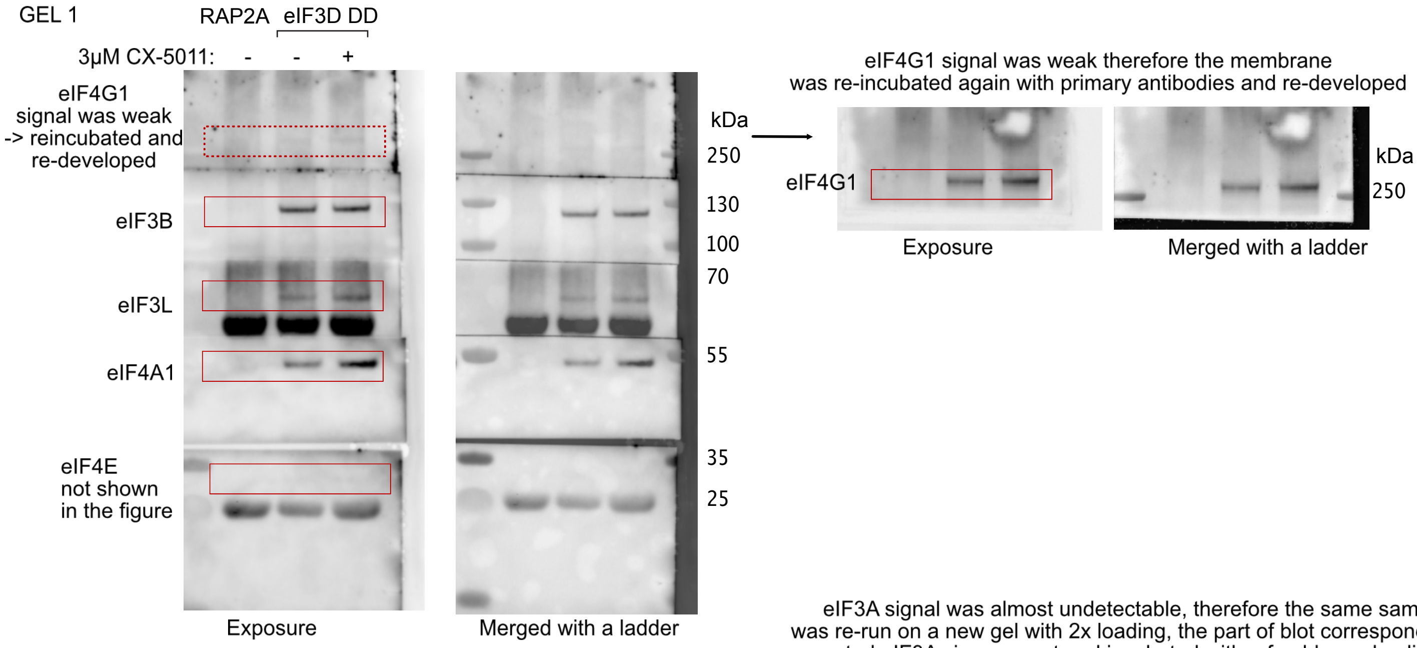

GEL 2 (loaded the same sample as at GEL1)

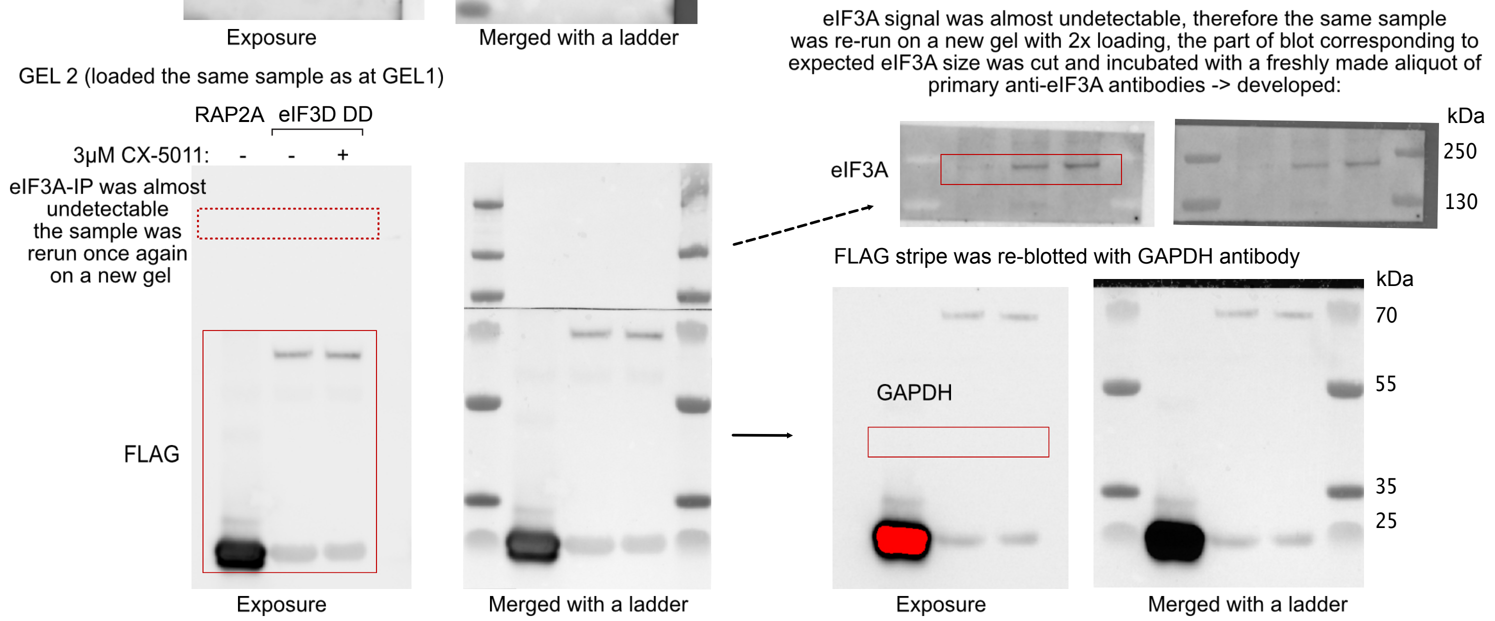

CORRESPONDING TO FIG S7E

Membrane directly after the western blot transfer was stained with ponceau S, and the image was acquired:

→ the same membrane was washed from ponceau S in PBS-T, the membrane was blocked and incubated with pan-pCK2 antibodies, developed:

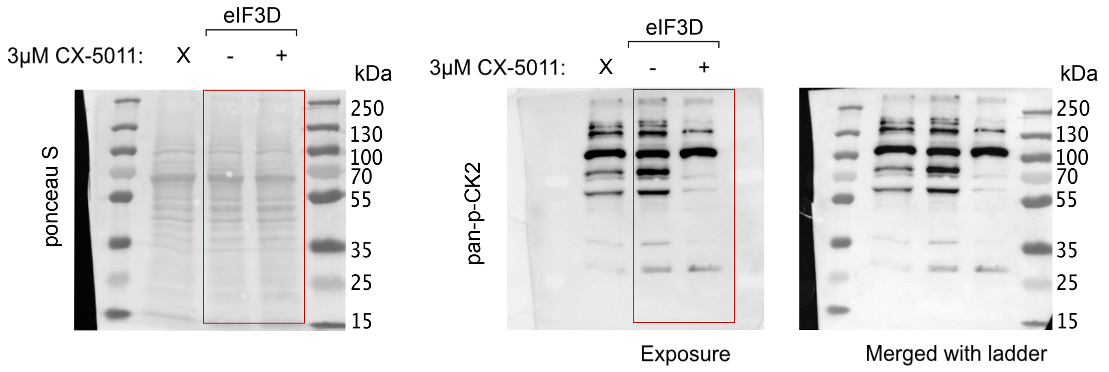

X-line → one more negative control (was not included in the final figure)
