# Supplementary material for: Signals from the niche promote distinct modes of translation initiation to control stem cell differentiation and renewal in the Drosophila testis
Source: PLoS Biol. 2025 Mar 11;23(3):e3003049. doi: 10.1371/journal.pbio.3003049 (PMC12136000; doi:10.1371/journal.pbio.3003049)
Supplement: Attachment — Submitted filename: Response to reviewers.docx [file pbio.3003049.s014.docx]

We would like to thank all the reviewers for their thoughtful comments. In addressing them, we believe we have substantially improved this manuscript and strengthened our conclusions. In particular:

- We have conducted new experiments in cell culture to show that a phospho-mimetic form of eIF3d can immunoprecipitate eIF4F subunits but is no longer sensitive to CK2 inhibitors, showing that the regulation of the eIF3-eIF4F interaction by CK2 depends on this phosphorylation.
- We have over-expressed a construct encoding the differentiation marker Eya, which contains endogenous UTRs, in CySCs. This experiment shows that elevated mRNA levels do not result in protein being present in stem cells. These data strengthen our conclusion that selective translation of mRNAs occurs in CySCs.
- We tested whether CK2 activity was regulated by JAK/STAT signalling at the level of expression. We find that expression of *CkIIα*, which encodes the catalytic subunit of CkII, is higher in CySCs than in differentiated cyst cells, and we show that this high expression depends on JAK/STAT pathway activity. This provides a link that was missing in the initial manuscript between niche signalling and CkII regulation.

In addition, we have added several controls and additional information, and edited the text in response to the comments, as detailed below. In particular, the discussion has been substantially revised, to address several points that were raised. The revised manuscript has two additional figures (one entirely new and another from splitting a previous figure) and four additional supplementary figures, as well as an additional supplementary table. We think the manuscript is much improved and hope the reviewers will agree.

-----------------------------------------
REVIEWS:


Reviewer #1: In this manuscript, Wang and colleagues report a biological phenomenon in which mRNA translation mediated by eIF4F-eIF3d1 is crucial for the self-renewal of somatic cyst stem cells (CySCs) of the Drosophila testes. Mechanistically, they demonstrate the importance of eIF3d1 phosphorylation by CK2, caused by the activation of JAK-STAT ligands from the testis niche. Importantly, eIF4F was found to be dispensable in differentiating cells, but is only required for the self-renewal of CySCs. Collectively, the authors identify a physiological role for translational control in determining stem cell fate. Although the finding does appear to demonstrate the importance of translational control in an in vivo setting, there is a stark lack of mechanistic details underlying the proposed specialized translation. Moreover, CK2-mediated mobilization of the eIF4F complex or phosphorylation-dependent control of eIF3 are already reported phenomena, and therefore lacks novelty.

We respectfully disagree with the reviewer about the novelty of our findings. While it was indeed known that CK2 phosphorylates eIF3d, this was shown in situations of nutrient deprivation or other stresses, during which eIF4F is inhibited (through the inactivation of mTor) (Lamper et al., 2020). Similarly, work showing that CK2 regulates eIF4F complex assembly showed that this function was mediated by mTor activity (Gandin et al., 2016). In CySC differentiation, we and others have previously shown that mTor activity is high, and necessary for differentiation (Amoyel et al., 2016; Senos Demarco et al., 2020; Yuen et al., 2021), indicating that mTor-dependent regulation of eIF4F activity cannot account for decreased translation rates during differentiation. Indeed, CySCs provide an excellent paradigm to understand how translation regulation can be uncoupled from mTor activity, especially in cells that use mTor signalling to mediate cell fate decisions rather than simple nutrient sensing.

Additionally, the previous work implied that the phosphorylation of eIF3d was important to regulate its cap binding ability in the absence of eIF4F activity (Lamper et al., 2020), but to our knowledge, there have been no studies examining the role of eIF3d phosphorylation in regulating translation initiation through other complexes, or in non-stressed conditions. Our findings reveal that CK2 controls the strength of the eIF4F interaction with eIF3, during normal homeostasis, through phosphorylation of eIF3d. Our data point to this interaction as a new regulatory point in the assembly of translation initiation complexes, something previously unappreciated in the literature which focuses mostly on the regulation of eIF4F or eIF2 activity.

Major Comments:

1.      In the text describing Figure 3A and B, authors claim that JAK/STAT signaling remains functional in mutant cells and that eIF4F knockdown does not greatly interfere with the translation of JAK/STAT signaling components, which are overreaching conclusions based solely on the presence/absence of Stat92E alone. To demonstrate this in greater detail, the authors should look for phospho-JAK or the relative expression of Stat92E target genes.

The antibody we used recognises Stat92e protein, which is normally stabilised only when signalling is active, and can be used as a readout for pathway activity (Rodrigues et al., 2012). When other labs use phospho-Stat antibody to label Drosophila testes, the pattern is indistinguishable from the one we observe with the Stat92e antibody (Xu et al., 2018; Zhang et al., 2017). To specifically address this point, we have examined expression of the JAK/STAT target *socs36e* by *in situ* hybridisation chain reaction upon knockdown of eIF4A. These data are shown in Figure S6 in the revised manuscript, and reported in the text on p. 9 and confirm our finding with the Stat92e antibody that JAK/STAT pathway activity is not reduced upon knockdown of eIF4F components. In addition, we have further examined *socs36e* expression upon knockdown of eIF3d1 and Ck2α and show similar results (Figures S6 and S8 in the revised manuscript).

2.      The authors have only demonstrated association of human eIF3D with eIF4F, which is already known as the authors mention in the text, in Figure 5G using HeLa cells. However, as the study is centered around Drosophila genetics, the authors should assess the interaction of Drosophila eIF3d1 with eIF4F by expressing them in human cells, or perform endogenous immunoprecipitation using S2 cells.

In our view, one of the strengths of our manuscript is that we used Drosophila genetics to infer a molecular relationship between eIF3d and eIF4F that was not known from the current literature (see above), and showed that this prediction could be validated in human cells. We turned to human cells in large part because of technical limitations that prevented us from assessing these molecular relationships in the Drosophila testis. These are 1) the limited availability of material – there are ~36 Zfh1-positive CySCs in each testis, making biochemical assays extremely difficult and 2) very few reagents available to study these proteins. For example, we tried to use several commercial antibodies against eIF3d, but none was able to recognise either endogenous or over-expressed Drosophila eIF3d1.

Given these limitations, we believe that showing this new relationship between eIF3d phosphorylation and the strength of interaction between eIF3 and eIF4F in human cells is a valuable addition. Expressing Drosophila eIF3d1 in human cells would not necessarily show a Drosophila-specific interaction, since it would incorporate into complexes with the human complexes, while experiments in S2 cells would not show interactions specific to CySCs of the testis, while having the disadvantage that there are fewer reagents available to work with. We therefore believe that complementing genetic assays in Drosophila with biochemical assays in HeLa cells is the best approach, benefiting from the advantages of both models. We have further strengthened the data showing that phosphorylation of eIF3d is important for the interaction with eIF4F with new experiments in the revised manuscript, which show that a phospho-mimetic form of eIF3d is no longer sensitive to inhibition by CK2 inhibitors (see below and Figure 6H,J in the revised manuscript). This again supports the conclusions we draw from genetic experiments, where the reduction of CySC numbers upon knockdown of CK2 is rescued by expression of phospho-mimetic eIF3d1, demonstrating the validity of our approach, and the conservation of our findings.

3.      The authors simply "infer" that eIF3d1 is phosphorylated, by using phosphomimetic mutants and based on previous reports that CK2 can phosphorylate eIF3, but do not provide any direct solid evidence (i.e. phospho-antibody/orthophosphate labeling) to conclude that eIF3d1 is truly undergoing phosphorylation-dependent control. This is especially important here, since the authors are claiming that CK2 phosphorylation-mediated effects on eIF3D cap-binding activity are not important under this setting.

We agree this is an important point, and this is one we have wanted to address ourselves. We have tried to raise an antibody specifically recognising the phosphorylated form of eIF3d1, unfortunately, the peptide we used was non-immunogenic, while several subsequent attempts failed to generate the required peptide. In the absence of a specific antibody, demonstrating phosphorylation in CySCs *in vivo* is extremely difficult. While in the past we have been able to use FACS to sort cells and isolate CySCs for RNAseq, we have only obtained ~10-20,000 cells per replica, which is suitable for RNA work, but several orders of magnitude lower than what is needed for biochemistry. This, along with the aim of extending our findings beyond Drosophila, is the reason we turned to cell culture to conduct biochemical experiments. We have edited the text of the manuscript, as well as the title, to temper statements that could not be directly supported.

4.      The decrease in binding between eIF3D and eIF4G1in Figure 5H is not very convincing, despite having quantified the difference in Figure 5I. To clarify the result the authors should perform either a dose/time course with the CK2 inhibitor, or assess the interaction following genetic depletion of CK2. Importantly, to firmly conclude that the eIF3D-eIF4G1 interaction is CK2-dependent, the authors should test whether the inhibitor has no effect on the interaction between eIF4G1 and eIF3D phosphomimetic mutant. Also worth noting is that the authors used a very broad CK2 inhibitor for their experiments, which has more than 100 substrates including other subunits of eIF3.

We thank the reviewer for this suggestion. In addressing this point, we have generated new results which significantly help strengthen our conclusions.
We expressed a phospho-mimetic form of eIF3d and show that, as predicted, the strength of eIF4F-eIF3d interaction is no longer sensitive to the CK2 inhibitor CX-5011. These results are presented in Figure 6H,J of the revised manuscript and described in the text on p.12. Notably, this agrees extremely well with the genetic result of a rescue of CySC numbers by eIF3d^DD^ expression in a CK2 knockdown (Fig. 6F). Regarding the inhibitor, we chose CX-5011 as it was shown to be effective in modulating eIF3d phosphorylation previously (Lamper et al., 2020). We agree with the reviewer that this is not an all-or-nothing change, but a modulation of the strength of interaction (~40% reduction), yet this is a reproducible change that is inhibited by expressing the phospho-mimetic form of eIF3d. We did repeat the experiment with another CK2 inhibitor, TBB, and observed a similar trend, although with much increased variability, leading to results just below the significance threshold. These results are shown below in Fig. R1.

Altogether, we thank the reviewer for this point, as we believe our data showing that phosphorylation of eIF3d modulates the strength of its interaction with eIF4F are substantially strengthened in the revised manuscript.

Figure R1: The CK2 inhibitor TBB produces similar changes of immunoprecipitation of eIF4F subunits with eIF3d as CX-5011. Top left: blot in which FLAG was immunoprecipitated in cells expressing a negative control (RAP2A) or eIF3d, in the absence or presence of the CK2 inhibitor TBB, and blotted with antibodies against subunits of eIF3 or eIF4F. The second blot shows the input without immunoprecipitation. Top right: two blots of FLAG-immunoprecipitated phospho-mimetic eIF3d, eIF3^DD^, and input, with antibodies against other initiation factors. Bottom left: blots showing all protein (Ponceau S) and a pan-CK2 substrate antibody, showing that TBB reduces staining. Bottom right: quantification of several repeats of the immunoprecipitations shown above, showing a trend in which TBB reduces immunoprecipitation of eIF4G1 and eIF4A1 by eIF3d, but not by eIF3d^DD^.

5.      The molecular link between JAK-STAT and CK2 remains missing. What do the authors hypothesize is happening? Although it has been reported that CK2 phosphorylates and activates JAK and therefore downstream JAK-STAT signaling, it does not explain how the expression of CKIIa rescues the phenotype of Stat92E mutant flies shown in Figure 6I. Are there other targets of CK2 that act to promote JAK-STAT activation? These ideas should be discussed in the Discussion.

We thank the reviewer for raising this point. This was also a question we wanted to answer, having identified CkII as epistatic to JAK/STAT in the initial manuscript. We have included new data in the revised manuscript (Figure 8) that we believe address this. Our genetic data placed CkII downstream of the effector transcription factor in the JAK/STAT signalling pathway, Stat92E (Fig 7E-F in the revised manuscript), indicating that CkII regulation of JAK/Hop is not the likely mechanism in this situation. Moreover, we did not observe changes in Stat92e levels or transcriptional activity upon manipulation of CkII (Fig. S8 in the revised manuscript), suggesting that CkII does not directly affect JAK/STAT activation. Instead, our data suggest that a transcriptional target of JAK/STAT pathway activity could regulate CkII.

Indeed, we tested whether CkII expression itself could be affected by JAK/STAT activity, and found that *CkIIα* expression decreased in *Stat92E^ts^* mutants. These results are reported in Fig. 8 in the revised manuscript, and indicate that Stat92E regulates *CkIIα*, although we have not determined whether this is a direct transcriptional induction. Thus, our new data suggest a link between JAK/STAT and CkII expression, which can help explain how CkII activity is restricted to the stem cells.

6.      The mechanism behind how differentiation genes (i.e. eya) are selectively translationally repressed in CySCs is completely absent in this manuscript. While it may be out of the scope of this manuscript to systematically profile the ribosome-protected fragments (RPFs), the authors should discuss the possible underlying reasons for this in the Discussion. For example, do there exist stem loop structures within the 5'UTR of eya that may display reduced affinity towards eIF3d1 binding so that it is rarely translated in CySCs?

We do indeed plan to conduct ribosome profiling in CySCs, but agree with the reviewer that this is out of the scope of this manuscript, which aimed to understand the regulatory mechanisms controlling translation modes. How this change in translation mode impacts specific targets is the next question we will address over the coming years. We have computationally analysed the *eya* 5’ UTR and there may be some stem loops consistent with those identified in prior reports, but we would want to validate this before making any strong claims. Identifying other transcripts regulated in this way through ribosome profiling would increase our confidence that this is indeed the mode of regulation. We have extensively rewritten the discussion, and now suggest possible mechanisms for selective translation in CySCs on page 16.

7.      Even though the authors focused on the CySCs as their model system, it would be interesting to assess the importance of eIF3d1 phosphorylation-mediated translational control in another Drosophila stem cell type to assess the universality of this regulation, again since the phosphorylation-dependent cap-binding switch does not appear to be important here. Could this finding then be generalized as a species-specific event?

We are unsure exactly what the reviewer is referring to in this comment. Our findings on the role of eIF3d1 phosphorylation indicate that it mediates the strength of interaction with the eIF4F complex, which we showed in human cells. Thus, the molecular regulation we identified is indeed generalisable as being similar in flies and humans. Regarding a role for this particular regulation in stem cells in general, we argue this is extremely unlikely. Although there is clearly a universal principle that translation must be regulated in stem cells to ensure appropriate fate determination, the precise mechanism by which this control is achieved will differ between the cell types. For example, in mouse, self-renewal of haematopoietic stem cells and satellite cells involves translational control, but the mechanisms described to date are different in each case (PI3K/4E-BP in HSCs (Signer et al., 2014; Signer et al., 2016) and phosphorylation of eIF2α in satellite cells (Zismanov et al., 2016)). This presumably reflects differences in the needs of the tissue to respond to specific environmental signals, or in the mRNAs that are specifically regulated which will be different in different stem cells.

Nonetheless, we examined whether the regulatory pathway we identified was also important in intestinal stem cells (ISCs) in Drosophila. We present these results below (Figure R2) but for the reasons stated above, we prefer not to include them in the manuscript. In brief, we knocked down CkII in ISCs using *esg-Gal4^ts^* and a titration control (UAS-LacZ), which led to mild disruptions to tissue morphology and a reduction in cell density and an accumulation of *esg*-positive doublets, presumably reflecting a differentiation defect in which the daughter cells of ISCs, enteroblasts, accumulate. We note that these phenotypes were much stronger without the titration control (not shown), indicating that CkII likely plays important functions in ISCs but that demonstrating this satisfactorily would require additional approaches. This accumulation of *esg-*positive doublets was reduced to control levels when the phospho-mimetic eIF3d1^DD^ was co-expressed, but not when the phospho-dead eIF3d1^NN^ was expressed. Thus, these results suggest that in ISCs and/or EBs, Ck2 regulation of eIF3d1 phosphorylation is also important, but that the targets might be different and be required for differentiation rather than self-renewal.

**Figure R2: epistatic relationship between eIF3d1 and CkII in the Drosophila intestine.** A-D: confocal images of the posterior midgut stained with DAPI. Intestinal stem cells and enteroblasts are labelled by GFP driven by *esg-Gal4*. Arrows point to doublets of GFP-positive cells. E: quantification of Esg-positive cell doublets, showing a trend towards an increase upon CkII knockdown, which is rescued by expression of phospho-mimetic eIF3d1^DD^, but not phospho-dead eIF3d1^NN^. F: quantification of the number of Esg-negative cells cells in a defined ROI (150x150μm or 22,500μm^2^) (ie the differentiated gut enterocyte and enteroendocrine cells), showing a trend towards decreased cell density upon CkII knockdown. These changes were much more apparent without the LacZ titration control for Gal4 activity (not shown), suggesting that the presence of an extra UAS impacts the efficiency of knockdown.

In sum, our data altogether argue that the regulatory interaction we have identified between Ck2 and eIF3d is conserved and important in many different cell types, including CySCs and ISCs in flies, and in HeLa cells, but that it may be used differently in each context. We expect that after we publish our manuscript, others will find the same regulatory interaction in other cell types and we are excited to see the variety of cell behaviours it may be important for.

8.      The authors' finding that eIF3d1 is crucial for global translation conflicts with a previous study (Szostak et al. Hrp48 and eIF3d contribute to msl-2 mRNA translational repression . Nucleic Acids Research, 2018; see Figure 5E) which showed only a "mild defect" in global translation upon eIF3d1 knockdown using Drosophila embryo extracts. The authors should compare and contrast the findings, and whether it could be attributed to the difference in cell types (i.e. embryo vs. testis)
We thank the reviewer for pointing this out. In fact, our data agree quite well with the findings from that paper, which showed a 25% decrease in translation in embryo extracts upon eIF3d1 knockdown, using ^35^S-methionine labelling. In CySCs, eIF3d1 knockdown resulted in 12% lower OPP incorporation, which is fairly similar to the 14% difference observed between CySCs in the first and second rows from the hub in control testes. The differences may be due to different efficiency of knockdown in different tissues or to the sensitivity of the assays themselves. We note that in our hands, the strongest reduction in OPP was induced by eIF4A knockdown (30%). We did note that some RNAis led to a complete absence of somatic cells (either CySCs or cyst cells, see Table S1), and one interpretation is that a too strong inhibition of translation is cell lethal. We have updated the manuscript to mention the range of decreased OPP labelling observed, and compared our findings to those of the study mentioned in the discussion on p.14.

Minor Comments:

1.      For the RNAi experiments, there are no control experiments showing the degree or efficiency of knockdown, for example by Western blot or FISH, making it difficult to conclude whether the observed phenotypes can be directly comparable.
In this manuscript, we conducted a screen of initiation factors by RNAi, screening over 30 genes.

We are confident that the results are likely to reflect true knockdown for several reasons: 1) for several genes, we observed the same phenotype with several RNAi lines (eg eIF4A, eIF4E1, eIF3b, and both Ck2 subunits), 2) We obtained similar results when knocking down several genes in the same translation initiation complex (eg all genes encoding subunits of eIF4F), giving us confidence in the specificity, and 3) for key genes we used mutant alleles where available (eg *eIF4A, eIF4E1, eIF3d1*) to reproduce the RNAi phenotypes.

We did not necessarily intend for the phenotypes to be directly compared, as geneticists, we are reluctant to discuss the relative strength of phenotypes obtained by RNAi. However, the main finding of the screen was that initiation factor knockdowns resulted in two classes of phenotype, with either testes containing ectopic Zfh1-positive cells or a loss of Zfh1-positive cells.

2.      The quantification strategy for immunohistochemistry data needs to be better explained in the Methods section, as the figures themselves do not always seem to reflect the dramatic changes illustrated on the quantified graphs.
We have updated the methods with a new section detailing how we quantified the data.

3.      There are numerous typos in the manuscript that deter readability. The authors should go through the text again carefully, also making sure that the Figures/Figure legends align properly with their descriptions within the Results.

We apologise for the typos and incorrect figure references. We have edited the text in the revised manuscript and hopefully have corrected these now.


Reviewer #2: This is an interesting paper that describes how the dynamic regulation of mRNA translation plays roles in both self-renewal and differentiation of cyst stem cells (CyCSs) within the Drosophila testis. OPP pulse labeling reveals subtle differences in global protein production between CySCs, their immediate daughters and their further differentiated progeny. RNAi knockdown experiments suggest eIF4F plays a specific role in CySCs, while the reduction of other tested translation factors resulted in the accumulation of ecotpic Zfh1 positive cells. The authors then present data that indicates eiF4F is epistatic to JAK/STAT. Interestingly, eiF4d appears to play a specific role in CySC self-renewal. Further work shows that CKII promotes the interaction between eIF4d and eIF4F. The paper ends with experiments showing that CKII mediates both autonomous and non-autonomous effects of niche signaling in CySCs.

While the ideas and results presented in the manuscript are interesting and worth pursuing, several weaknesses significantly dampened enthusiasm for the paper. Overall the results appear preliminary and should be further developed before publication in PLoS Biology.

Major:
The authors conclude that eIF4F knockdown results in specific effects of CySC self-renewal that are different from other translation factors. However no controls/data are presented for the expression of these genes within the CySC lineage and after RNAi knockdown. Are these phenotypes rescuable by non-targeted transgenes?
We used multiple RNAi lines targeting different genes in the same complex and obtained similar phenotypes. Importantly, however, we validated the RNAi phenotypes with mutant alleles of *eIF4A* and *eIF4E*, and showed that CySCs mutant for either subunit do not self-renew and differentiate.

Unclear how the OPP immunofluorescence was quantified. Were whole cells used or regions of interest within the selected cells? OPP labeling can be variable from sample to sample, so care should be taken when evaluating results.
We have updated the methods to better explain the quantifications of OPP levels. In brief, we agree with the reviewer that OPP can vary between samples, so in all measurements, we have normalised OPP levels in CySCs (measured individually using Dlg staining to outline cells), either to the hub, or to GSCs (depending on the manipulations, as for instance, JAK/STAT loss of function would be expected to affect GSCs as well as CySCs).

eIF4F and the other translation factors are all essential genes, and it seems unlikely that severe knockdown would allow cells to survive. This should be quantified. A slightly amended model to the one being proposed by the authors is that CySCs produce and contain all the protein needed for differentiation before their daughters begin to move away from the niche.

The reviewer raises an intriguing point. However, the model proposed would not fully explain our data, since differentiation factors (eg Eya) are never observed in CySCs, but can be detected in *eIF4A* or *eIF4E1* mutant cyst cells. Since mutant clone generation requires cell division and since only CySCs divide in the cyst cell lineage, the mutant cells observed are derived from a CySC division and therefore have been able to synthesise new Eya protein. Indeed, the new data we provide in response to the comment by Reviewer 4 below show that expressing *eya* mRNA does not lead to expression of Eya protein in CySCs, indicating that there is additional regulation through translational control.

Regarding the survival of eIF4F-deficient cells, previous work has established that the *eIF4A* alleles we used are strong loss-of-function alleles, but that they are not cell lethal. Animals homozygous mutant for these alleles do not survive (Galloni and Edgar, 1999). Similarly, the *eIF4E1* mutant allele we used results in lethality after several days of development (Lachance et al., 2002), indicating that cells can survive without eIF4E1, although development is impaired.

The authors rule out cell death as a possible explanation for the loss of cells based on negative results from p35 over-expression. No controls for the effectiveness of p35 in suppressing cell death in this system are given. In addition, no assays to detect cell death in its many forms are presented. The authors should provide data on these points.
We thank the reviewer for raising this point. We have added new data in Figure S3 in the revised manuscript showing that expressing p35 results in a strong reduction in testes containing any activated Dcp-1-positive cells, indicating that it is effective in suppressing cell death. We further show Dcp-1 stains in various genotypes (Fig. S3, S6).

The authors present data on the effects of different eIF4d transgenes, but the expression of each transgene in the experiments should be evaluated before firm conclusions can be made.
We presume the reviewer refers to the eIF3d1 transgenes that we use in the manuscript. We have attempted to use antibodies against mammalian eIF3d to assess expression of the transgenes; however, these antibodies do not recognise either endogenous eIF3d or over-expressed eIF3d. Without a validated antibody, it is difficult to evaluate expression as unfortunately our transgenes did not have a peptide tag. We considered using *in situ* hybridisation to assess expression *in vivo*, however, we do not think this would address the reviewer’s concern as it would not show protein expression.

Nonetheless, we are confident the transgenes are expressed, since the phospho-mimetic and wildtype constructs can rescue CySCs upon Ck2 knockdown, and the phospho-dead construct produces a dominant-negative effect on CySC self-renewal when expressed on its own. In addition, we provide new data in the revised manuscript (Figure S7D) showing that expression of phospho-dead eIF3d1 reduces OPP incorporation, again indicating that it can act as a dominant negative. Thus this transgene can drive sufficient levels of expression to interfere with endogenous eIF3d1. We note that all transgenes are inserted in the same genomic locus, and therefore should drive similar levels of expression.

The authors propose a provocative model whereby eIF4d activity results in the translation of specific messages. However, the identity of those messages remains unknown and thus the model is untested. It is unclear whether any target mRNAs show changes in translation through direct regulation by these factors.
As mentioned above (reviewer 1, point 6), we intend to conduct ribosome profiling to identify mRNAs that are specifically targeted by eIF4F activity. This lies out of the scope of the current work, but we have included a discussion of the possible mechanisms by which this regulation could be achieved on p.16, as suggested by Reviewer 1. A likely target is *eya*, which is present but not translated in CySCs, as we show in Figure 3 of the revised manuscript, and is regulated post-transcriptionally as our new data show that over-expressing *eya* is not sufficient to cause Eya protein accumulation in CySCs. Identifying further targets regulated in this way will allow us to shed light on the mechanism by determining if they share sequence or structural features.

The authors should block CySC lineage differentiation independent from expanding JAK/STAT signaling, if possible and evaluate OPP labeling. Experiments like this one is critical for testing the idea that niche signaling and not differentiation per se regulates protein synthesis within this lineage.
We thank the reviewer for this suggestion. We had previously shown that CySCs differentiation could be blocked through knocking down the Retinoblastoma homologue Rbf, and that this results in expansion of CySCs independently of niche signals (Sainz de la Maza et al., 2022). We have therefore used this paradigm to measure OPP levels in ectopic CySCs compared to CySCs adjacent to the hub, and find significantly lower OPP in ectopic Zfh1-positive cells away from the hub. These results are presented in Figure S1 in the revised manuscript and described in the results on p.5. We are grateful to the reviewer for suggesting a way to demonstrate further that translation changes are due to niche signals.

Minor:
Do manipulations expected to modulate protein synthesis (mTOR activation etc) result in changes in OPP labeling?
The reviewer raises an important point, and indeed we believe that the relationship between protein synthesis and mTOR in CySCs is worth exploring, and we are currently preparing a separate manuscript on this question. We have generated substantial data to show that Insulin and mTOR do affect translation but that they do not influence cell fate through this regulation. Indeed, we believe these data allow us to show that the global changes in translation rates can be uncoupled from the regulation of specific transcripts, indicating that target specificity does not come from Insulin/mTOR-dependent pathways. Over-activating Insulin signalling (by expressing the PI3K catalytic subunit dp110) results in significantly higher OPP incorporation in CySCs:

Notably, Tor pathway loss-of-function results in the opposite phenotype than that of eIF4F inactivation: *tor* mutant clones cannot differentiate (Amoyel et al., 2016). This suggests that in the CySCs, Tor regulation of eIF4F is not the mechanism by which translation specificity is achieved. Indeed, we have data to show that Tor’s known effectors in translation control, 4E-BP (encoded by *thor*) and S6k, do not influence CySC fate. We have also generated double mutants for *eIF4A* and *tor*. Where *eIF4A* mutant CySCs differentiate rapidly, and *tor* mutant CySCs cannot differentiate, double mutants produce an intermediate phenotype with cells often expressing markers of both CySCs and differentiated cyst cells. We interpret these results to mean that *eIF4A* and *tor* are independent, and therefore that Tor does not function through the regulation of translation. Altogether, our data make a compelling case that both CkII and mTOR can regulate global translation rates, but that the specificity in translated targets is achieved through the CkII-eIF3d1-dependent regulation we identify here, not through mTOR.

Several groups have investigated dynamic mRNA translation in the female germline, but few if any of these references are cited or discussed.
We apologise for this omission. We are aware of the literature in the female germline, but chose to keep the discussion short initially. We have substantially revised the discussion to mention the female germline and have compared our findings in the male gonadal soma to the published work in the female germline.


Reviewer #3: This manuscript by Wang, et al. provides an interesting and novel perspective on how the stem cell niche microenvironment contributes to stem cell fate regulation through influencing translational control. The goal of this study was to identify and describe a mechanism of translational regulation using somatic cyst stem cells (CySCs) and their differentiated cyst cell progeny from the well-studied Drosophila male testis. The authors studied translation initiation factors and found different requirements for translation initiation cap binding proteins in CySCs versus differentiated cyst cells. They dissect this mechanism further, pinpointing a phosphorylation event that regulates the interaction between cap binding proteins that promotes different translation programs. This gives cells the ability to rapidly respond to environmental signals by switching the translation mode. Altogether, this study adds an interesting perspective on how translational control
provides a tunable response mechanism for protein expression that influences cell fate decisions. The findings are relevant to many biological situations that require rapid adaptation including development, tissue homeostasis, and diseased states.
The experimental strategies described in this study are well-designed, utilize orthogonal approaches and appropriate controls, and are presented logically throughout this well-written manuscript. The authors further provide adequate background information at appropriate places such that a non-expert can understand and appreciate the topic. Despite this, there are a few questions and addressing these would help clarify some aspects.
Experimental or data interpretation considerations:
1.      I am curious as to how a general shift in temperature shift translational rates. If the data in Fig 1H were to be plotted normalized to the WT control in Fig 1B, what would that look like?

Unfortunately it is difficult to compare OPP levels across samples, as there is some variability from experiment to experiment, as noted above by Reviewer 2. This is why we used an internal control (hub or GSCs) to normalise OPP levels and enable comparisons of OPP signal in CySCs. However, this method would not allow us to conclude about general changes occurring with changes in temperature since the cells we use for normalisation would also be affected.

2.      The model in Fig. 7 indicates a steep change of JAK-STAT in CySC vs. cyst cell with the Stat92E to be ON in CySC but entirely OFF in cyst cell, but immunostaining using anti-Stat92E in the control in Fig. 3A does not seem to show this pattern?
In general we do observe the pattern described here, where JAK/STAT activity is on in CySCs and off in differentiating cyst cells (eg Fig. S8A in the revised manuscript). In clonal experiments, clones are not always in plane with the hub and can be either above or below in the Z-stack, meaning that we had fewer images to choose from when selecting an image from this panel. The image referred to by the reviewer (now Fig. 4A of the revised manuscript) was not representative of the stainings we usually observe and has been replaced.

3.      How does the JAK-STAT signaling control the activity of the CKII kinase activity or expression level or both?
This is an excellent question and one which we also wanted to answer. See also response to Reviewer 1, point 5 above, but in brief, we have added new data to the revised manuscript (Figure 8), showing expression of *Ck2α* is higher in CySCs than differentiated cyst cells and decreases upon JAK/STAT loss-of-function. This suggests that the activity is controlled at the level of expression as suggested by the reviewer.

4.      Immunoprecipitations were done using Hela cells, how can these cells recapitulate the protein-protein interactions in the somatic gonadal cells in fly testis?
While we agree that Drosophila cyst cells and HeLa cells may behave differently, we believe that a great strength of our work is the fact that our genetic analysis led us to make a novel prediction regarding the biochemical function of a protein, and that this prediction was borne out in HeLa cells. In this sense, it is not appropriate to regard the immunoprecipitation data as separate from the rest of the manuscript, since it was the product of and only came about because of the genetics. We could have tested the model that our genetic data led to using another cell type, such as S2 cells, however we chose to use a human cell line as this would show conservation of the mechanism we propose and make our work relevant beyond the Drosophila community. In our view using two complementary approaches, genetics in Drosophila and biochemistry in a human cell line, provides strong validation for a new model of translation regulation. In fact, it would be extremely difficult, if not impossible, to conduct these experiments using CySCs, of which there are ~36 per testis, meaning that we cannot obtain sufficient material for immunoprecipitation. In addition to the reduced number of reagents available, this means that the testis is simply not a good model for biochemical experiments. However, the evidence from Drosophila genetic data through knock downs and epistasis experiments is consistent with the biochemical data from HeLa cells, together building a model for the regulation of eIF3-eIF4F interactions by phosphorylation of eIF3d.

Minor notes:
1.      Please include the microscope and software used for imaging in the methods.
We have updated the methods section in the revised manuscript to include this information.

2.      Additional details on quantification of OPP should be present in the methods:

a.      Was this done in Fiji or Imaris?
b.      Are these measurements done on a single Z-slice or from the entire cell?
c.      What was used for the normalization? (it was mentioned in the figure legend, not the methods that the hub was used)

We apologise for these omissions – all this information has been added to the updated methods section. Regarding normalisation, we have used both hub and GSCs for normalisation, depending on the experiment, as some experiments could affect those cell types too (eg JAK/STAT loss of function using *Stat92e^ts^* would be expected to affect GSCs as well as CySCs). This information is now in the figure legends and the rationale behind the choice of cell to normalise is explained in the methods.

3.      Double check that the stock information for all fly lines used in the study are in the methods. Fly lines used for the clonal analysis seem to be missing:

a.      Example: Supplemental Figure 1P shows RFP clones. What is the membrane bound protein that is tagged with RFP to indicate these clones? This seems to be a different fly line than what was used to make the eiF4A clones in the same figure. There, both the positive and negative clones have a nuclear signal.

We apologise for the confusion, we used several different stocks to make clones. The figure (now split between Fig. S2 and S4) showed positively-labelled clones in which the nucleus of labelled cells was marked with UAS-nlsGFP (Fig. S2A-B), as well as negatively-labelled clones in which the signal is both cytoplasmic and nuclear (Fig. S2I-L) – but note that clones are marked by absence of GFP in this case. For the example cited by the reviewer (now Figure S4A-B), we used a separate stock to positively label cell membranes with UAS-LifeAct-mCherry, so that we could visualise the membranes of mutant cells and assess their encapsulation of germ cells. We have updated the methods to explain the stocks used, and added a new supplementary table (Table S2) to detail the experimental genotypes used in each figure.

4.      To a non-expert, the localization pattern of ImpL2-GFP is unclear in Figure 2L-2O. Is it cytoplasmic, membrane-bound, nuclear?
a.      Is the ImpL4-GFP a transgenic or knock-in fly line?

ImpL2-GFP is a knock-in line, in which a MiMIC element inserted into the genomic *ImpL2* locus, in the 5’ UTR of the gene, was replaced with a GFP-containing cassette by recombination-mediated cassette exchange (Venken et al., 2011). While RMCE is used to generate protein traps, since this is an insertion in the 5’ UTR, the resultant expression is cytoplasmic (whereas ImpL2 is a secreted protein, and antibody staining reveals a punctate pattern on the surface of CySCs (Amoyel et al., 2016)). Here we just used ImpL2-GFP to outline somatic cells, as GFP is easier to stain after *in situ* hybridisation than other epitopes.
b.      The signal is quite weak, thus indicating ImpL2's localization within CySCs by drawing the boundaries of a single cell would be helpful.

We have outlined a CySC in the figure (now Figure 3 in the revised manuscript) and included an inset of an enlarged CySCs to show *eya* expression in CySCs more clearly.
c.      A zoom-in of a single cell will clarify true eya RNA signal looks like.
See above, an inset is included in Figure 3.

5.      For Figures 4A and 5A-C, is there an additional (grey) channel that is not labelled?

There was no additional channel in grey. We think the cyan channel was a little weak in some images and may have appeared grey.

6.      VDRC RNAi: Are these dsRNA or shRNA?
Most of the VDRC lines we used are from the GD or KK libraries, so encode long dsRNAs. We have updated the description of the stocks we use in the methods section to clarify which library each is from.


Reviewer #4: In this study, the authors have described an original model to elucidate how cell identity transitions occur in Drosophila testis cyst stem cells. Initially, they demonstrate that global translation rates are high in CySCs and decrease during differentiation in a JAK/STAT-dependent manner. However, by knocking down different translation initiation factors, they show that the global rate of translation does not determine cell fate. Instead, they identify two initiation factors, eIF4F and eIF3d1, as crucial for CySCs' self-renewal and fate determination.

Indeed, the authors find that eIF4F and eIF3d1 are necessary for CySCs' self-renewal. They observe that eIF4F acts downstream of JAK/STAT activity, and they demonstrate that Casein Kinase II phosphorylation of eIF3d1 is crucial in this pathway. Overall, they suggest that CySCs have a specific translational program that requires the eIF4F complex regulated by eIF3d1 phosphorylation. According to the proposed model, stem cells possess mRNAs encoding self-renewal and differentiation factors that can be selectively translated by switching a translational program, thereby determining cell fate.

Overall, there is much to commend in this manuscript. Initial knock-down experiments using RNAi are supported by genetic mutants for the most significant genes, using both positively and negatively marked genetic clones. The loss of stem cells in specific mutant conditions is demonstrated to result from differentiation rather than cell death (p35 experiments). Moreover, genetic experiments and interactions are nicely complemented by molecular experiments showing that the phosphorylation of eIF3d1 could impact its molecular partners. Thus, this manuscript extends beyond describing a genetic pathway important for stem cell self-renewal and differentiation. Furthermore, the manuscript is candid in describing unexpected results that contradict the initial assumptions of the authors.

However, several crucial points need addressing to validate some of their results and test the authors' model. Specifically, the authors place translational regulation at the core of their model to switch between self-renewal and differentiation. Ideally, one would over-express an RNA sufficient for differentiation in stem cells with its endogenous translational regulatory regions (5' and 3' UTR). Their model predicts that this should not be translated and thus should not lead to stem cell differentiation. Then, repeating the same experiment with ubiquitous 5' and 3' UTR should result in translation and in stem cell loss. Alternatively, the authors could conduct the reverse experiment with an RNA sufficient for self-renewal and express it with and without its UTRs in differentiating somatic cells.
This is an excellent suggestion and one partially overlapping with point 1 below. Unlike the germline, however, we are not aware of a factor that drives differentiation when over-expressed. It was previously shown that over-expressing Eya and its transcriptional partner Sine Oculis (So) does not lead to precocious differentiation (Fabrizio et al., 2003). Notably that study did not test whether the over-expressed constructs led to protein expression, and we have now tested this as part of the revisions for our manuscript (see below).

Major comments:
1)      When testing the importance of translational control, some key results are presented in Figure 2L-O, where the authors compare the expression of eya mRNA and Eya protein. However, the images lack clarity, making it difficult to discern cell locations, especially in close-up captions. For instance, in Figure 2M-M', eya mRNA is detected in both CySCs (dashed arrow) and differentiating cells (arrowhead), while Eya protein is only observed in differentiating cells (Figure 2L' arrowhead). Nevertheless, the levels of eya mRNA appear markedly different between stem cells (low) and differentiating cells (high), raising doubts about whether the difference in protein levels might partly result from varying levels of transcription. To address this concern, it would be important to overexpress eya mRNA in stem cells and observe any resulting appearance of Eya protein.

We thank the reviewer for this careful analysis and for the experimental suggestion. We have over-expressed *eya* in CySCs using tj-Gal4, these results are shown in Fig. 3E in the revised manuscript. In these experiments, we observed elevated *eya* mRNA levels in CySCs and only occasional Eya protein expression. Thus, we argue that it is not simply levels of *eya* expression that determine whether the protein will be present or not.

We have further provided higher magnification images clearly showing *eya* mRNA in CySCs. As noted by the reviewer, endogenous *eya* mRNA levels are indeed lower in CySCs than in differentiated cyst cells. This has been described in previous analyses of sequencing data comparing CySCs and cyst cells; there is a large increase in *eya* levels in differentiated cyst cells, but *eya* mRNA is detected in CysCs which were sorted by FACS (see figure below, taken from our recent work (Sainz de la Maza et al., 2022)). The *in situ* hybridisation data we present validate these sequencing results in vivo, and show that these *eya* transcripts are present even in cells that contact the hub.

Our work does not seek to argue that transcriptional regulation of cell fate does not occur; it does. Instead, we argue here that there is an additional layer of control through translation, since at least some factors are expressed at the RNA level in both CySCs and cyst cells. The experiment described above, to express *eya* mRNA and assess protein production has helped us demonstrate this more clearly, and we thank the reviewer for the suggestion which we believe has improved our manuscript.

2)      The model posits a switch of translation factors that determine cell fate. It would be important to show, under wild-type conditions, the endogenous expression of key eIF factors through immunofluorescence or tagged proteins. Additionally, it would be instructive to investigate the behavior of these eIF factors in the presence of JAK/STAT mutations and during upd upregulation to determine if their expression profiles and localization align with the model proposed in this manuscript.
We have examined expression of initiation factors using available protein trap lines. Most are ubiquitously expressed, as shown below and in previous work by others (Ghosh and Lasko, 2015). Analysis of RNAseq data reveals similar ubiquitous expression of most translation initiation factors (not shown). Since levels are not higher/lower in CySCs than differentiating cyst cells, it is unlikely that they are regulated at the transcriptional level by JAK/STAT signalling. However, we note that our model does not imply a change in expression of initiation factors, merely of their activity. Our data suggest that this activity is modulated by phosphorylation of eIF3d1, in a manner that depends on the kinase Ck2. We did not think that adding these data showing lack of regulation of expression levels of initiation factors would benefit the manuscript, however we can include these if the reviewer deems it necessary.

Figure R3: Endogenous protein trap for eIF4G1 shows ubiquitous and uniform expression in the testis. The arrow points to a CySC and the dashed arrow to a differentiated cyst cell many cell diameters away from the hub (indicated with an asterisk).

3)      In Fig 3A,B, and B'': how do the authors select cells to normalize fluorescence? In 3B'', a cell on the right side is chosen (arrowhead), but why not select the one on the left side with higher expression?
We apologise for the confusion. We have updated the methods to clarify the quantifications. In brief, we counted all clearly identifiable CySCs in the same Z-plane and used the mean of control cells to normalise fluorescence for the mutant cells. We chose to highlight one example in the figure but this does not imply it was the only cell counted.

4)      The authors show that eIF4F functions downstream of JAK/STAT in CySC self-renewal. In Figure 3E, it is observed that eIF4G1 mutation rescues the upd overexpression. Do other eIF4 factors also rescue the phenotype? Can an eIF3d mutation rescue upd overexpression too?

We thank the reviewer for pointing this out. We have now assessed whether knockdown of other eIF4F subunits or eIF3d1 could suppress the phenotype of JAK/STAT gain of function and find that knockdown of eIF4A and eIF3d1 could indeed partially suppress, as assessed by the presence of Eya-positive cyst cells. For technical reasons we could not assess eIF4E1 as the only RNAi line we had was on the same chromosome as UAS-Upd. These results are now shown in Fig. S6 in the revised manuscript.

5)      In Fig. 5F, the expression levels of wt, NN, and DD eIF3d could differ in the testis, potentially explaining the varying rescue profiles. Do they exhibit similar expression levels?
See response to reviewer 2 above. All constructs are inserted in the same genomic locus using φC31 integration at attP40, so expression levels should be comparable. We have been unable to detect eIF3d expression in flies using available antibodies. However, we are confident that the constructs are expressed, as eIF3d1^WT^ and eIF3d1^DD^ can rescue Ck2 knockdown, while eIF3d1^NN^ behaves like a dominant-negative when expressed in a wild type background. We have strengthened these data by showing that eIF3d1^NN^ expression results in reduced OPP incorporation (Fig. S7D), showing that its expression directly impacts translation rates.

6)      The experiments depicted in Figure 5 are conducted in human HeLa cells. Is eIF3d phosphorylated in Drosophila testis?
See response to reviewer 1, point #2 above. We have so far failed to generate an antibody specifically recognising phosphorylated eIF3d1. Without this, assessing eIF3d1 phosphorylation in the testis is challenging, simply because we cannot obtain the amount of material needed for biochemical assays. We have edited the text in various places to avoid directly implying that eIF3d1 is phosphorylated in the testis.

7)      It would be interesting to test whether overexpression of eIF4F could rescue ckII mutants, suggesting that critical levels of eIF4F are crucial for self-renewal, independent of eIF3d phosphorylation.

In principle we agree that this would be an interesting hypothesis to test. However, it could be experimentally challenging, as eIF4F has 3 subunits (and all are genetically required). We could try over-expressing subunits independently, but since we do not know if one particular subunit is limiting or if levels of the whole complex are important, we could mis-interpret negative data from this experiment. We would not be able to express all the subunits together due to lack of space on the chromosomes (3 UAS transgenes, plus the Gal4 and the Ck2 RNAi), and would observe much reduced effects of the knockdown due to Gal4 titration, making this experiment extremely unlikely to provide interpretable information in practice.

8)      In Fig. 6, the authors demonstrate that overexpression of CKIIα can rescue stat92 mutants. Can the expression of phosphomimetic eIF3d rescue stat92 mutants compared to phosphor-dead or wild-type versions?
We have attempted this experiment. However, the crosses and the offspring for this genotype are extremely sick, meaning that we have been unable to observe sufficient numbers of testes to have confidence in our results.

9)      In Figure 2B, it is unclear whether the different mutants are sterile.
This screen aimed to uncover translation factors that affected early fate decisions in the somatic lineage of the testis. We did not assessed fertility for the screen, as fertility could be affected by many factors, including much later events than the stem cell differentiation phenotype we scored. Instead, we simply used the presence of Zfh1-positive cells to indicate fate defects. We note that we induced knockdown in adults by shifting *tj^ts^-Gal4* flies to the restrictive temperature for Gal80^ts^ 1-3 days after eclosion, so that fertility might not be fully affected as there are most likely spermatids that result from earlier differentiation.

Minor comments:

1.      In the introduction, authors say "… used as a marker for CySCs (Inaba et al. 2011)…". This article focuses on String and cdc25, it could be replaced by Tulina Matsunis Science 2001 for example?

This sentence referred specifically to the use of Zfh1 as a marker for CySCs. The 2001 *Science* paper did not mention Zfh1, since it was only shown to be expressed in CySCs by Leatherman and Dinardo in 2008. The Inaba paper was one of the first to use Zfh1 as a marker to count CySC numbers. We cited the Tulina and Matunis paper in the previous sentence, where we describe the activation of JAK/STAT signalling in stem cells surrounding the hub.

2.      In the introduction, authors explain the model and mention Fig1A as support. This figure needs labels for CySCs, GSCs, 1st row and 2nd row to better understand the rest of the figures.
We have updated the figure (now Figure 1B) with legends. We thank the reviewer for helping to make this manuscript more accessible to readers.

3.      In the introduction, authors explain translation initiation without a Figure Support. The scheme of Fig.2A could be included in FIG.1 for better understanding of the model they suggest from the beginning.
We have moved this diagram to Figure 1A in the revised manuscript and cited it in the introduction, we agree this helps readers and thank the reviewer for the suggestion.

4.      In the introduction, authors could consider to mention Ghosh Lasko Plos One 2015 about eIF4E, eIF4E-3, eIF4G and eIF4G2 during spermatogenesis, and Shao. et al. Developement 2023, about eIF4E5 which is important for spermatogenesis.
We apologise for the omission and have cited these and other papers examining the role of eIF4F subunit paralogues in the testis in the revised introduction. We had wanted to avoid confusion by discussing the germline, as the presence of germ cell-specific paralogues and other germ cell-specific translational control mechanisms is maybe less relevant to somatic cell types, but we realise that this resulted in omitting several papers on initiation factors in the testis, which we have now corrected.

5.      The quantifications are explained in each legend and only sometimes in the main text. They should be explained systematically also in the main text since the differences are sometimes subtle and without the quantification technique is less convincing.
We have updated the text in the revised manuscript to include a section in the methods explaining the quantifications.

6.      Fig.1G': the light violet dashed 2nd row lines are difficult to observe.
We have changed the colours to make them easier to see.

7.      Results: Overexpression of upd results in higher levels of OPP incorporation in Zfh1 positive cells (Fig.1I): Could zfh1 impact ribosome biogenesis explaining the increase of translation?
This is an interesting possibility. Our data suggest that eIF4F is required downstream of JAK/STAT, but it is likely that JAK/STAT also affects other aspects of translation such as ribosome biogenesis. We note that we do not observe any obvious increases in Fibrillarin expression in CySCs compared to cyst cells (unpublished observations and also visible in images from (Sanchez et al., 2016)). Fibrillarin is often used as a marker of ribosome biogenesis, suggesting that if there is increased biogenesis in CySCs upon JAK/STAT activation (or expression of its targets such as Zfh1), it may be only a small effect.

8.      Results Fig S1G-J, the label eiF4E1 is difficult to see.
We have moved the legend to make it clearer.

9.      Results Fig S1Q, Vasa perinuclear staining seems affected compared to the control. What happens in eiF4E?
We do not think the Vasa staining was consistently different, merely that the antibody stain was less good in the image we show. We have found considerable variability when using the DSHB anti-Vasa antibody. In this experiment, since we wanted to show a clone, we chose an image in which the staining was maybe not the best. We have included a new example in the figure (now Figure S4 in the revised manuscript), in which the Vasa staining is more similar to control. We chose to keep the previous example as well, as it showed two mutant cyst cells.

10.     Results Fig.2GH, CySCs continue to self-renew, is this independent of Jak/Stat? Is it possible that eiF3H inhibits zfh1 transcription?
We have not assessed JAK/STAT activity in all the knockdown conditions; however, when we have, we have not observed any ectopic JAK/STAT activity, suggesting that the ectopic CySCs are indeed independent of JAK/STAT. However, we focused our study on the factors required for self-renewal as these were the most likely ones to be regulated by niche signalling. Regarding *zfh1* transcription downstream of translation initiation factors, since many factors (ie all eIF3 subunits except eIF3d, as well as eIF1, 2, 5 and 1A) result in ectopic Zfh1-positive cells, and since this is not a full block on differentiation, with Eya-positive cells often intermingled with the ectopic CySC-like cells, we do not think this is a specific effect of a particular factor on transcription, but most likely the induction of a stress response such that translation factors indirectly affect gene expression.

11.     Authors write " …phospho-mimetic eIF3d1 had no effect on CySC numbers (Fig.S3B)": this is wrong, it should be S3C.
We apologise for this mistake, the figure reference has changed in the revised manuscript (Fig. S7C) and we have tried to be careful in ensuring all figure references are now correct.

12.     Authors write "However, expression of this construct did not affect CySC numbers (Fig.S3C)". This is wrong, it should be S3D.
As above, we apologise for the mistake and have updated figure references in the revised manuscript.

13.     Authors write "…with a pan phosphor CK2 substrate antibody (Fig.S3D)": it should be S3B.
As above, we apologise for the mistake and have updated figure references in the revised manuscript.

14.     In the graphical abstract/working model (Fig.7), homogenize with the scheme of Fig1 by labelling the important cells of the study directly in the figure: CySCs, 1st row, 2nd row, etc.

We have updated the figure to make the manuscript more coherent as suggested by the reviewer. We agree this will improve the overall readability of our manuscript.

References

**Amoyel, M., Hillion, K. H., Margolis, S. R. and Bach, E. A.** (2016). Somatic stem cell differentiation is regulated by PI3K/Tor signaling in response to local cues. *Development* **143**, 3914-3925.

**Fabrizio, J. J., Boyle, M. and DiNardo, S.** (2003). A somatic role for eyes absent (eya) and sine oculis (so) in drosophila spermatocyte development. *Developmental Biology* **258**, 117-128.

**Galloni, M. and Edgar, B. A.** (1999). Cell-autonomous and non-autonomous growth-defective mutants of Drosophila melanogaster. *Development* **126**, 2365-2375.

**Gandin, V., Masvidal, L., Cargnello, M., Gyenis, L., McLaughlan, S., Cai, Y., Tenkerian, C., Morita, M., Balanathan, P., Jean-Jean, O., et al.** (2016). mTORC1 and CK2 coordinate ternary and eIF4F complex assembly. *Nat Commun* **7**, 11127.

**Ghosh, S. and Lasko, P.** (2015). Loss-of-function analysis reveals distinct requirements of the translation initiation factors eIF4E, eIF4E-3, eIF4G and eIF4G2 in Drosophila spermatogenesis. *PLoS One* **10**, e0122519.

**Lachance, P. E., Miron, M., Raught, B., Sonenberg, N. and Lasko, P.** (2002). Phosphorylation of eukaryotic translation initiation factor 4E is critical for growth. *Mol Cell Biol* **22**, 1656-1663.

**Lamper, A. M., Fleming, R. H., Ladd, K. M. and Lee, A. S. Y.** (2020). A phosphorylation-regulated eIF3d translation switch mediates cellular adaptation to metabolic stress. *Science* **370**, 853-856.

**Rodrigues, A. B., Zoranovic, T., Ayala-Camargo, A., Grewal, S., Reyes-Robles, T., Krasny, M., Wu, D. C., Johnston, L. A. and Bach, E. A.** (2012). Activated STAT regulates growth and induces competitive interactions independently of Myc, Yorkie, Wingless and ribosome biogenesis. *Development* **139**, 4051-4061.

**Sainz de la Maza, D., Hof-Michel, S., Phillimore, L., Bokel, C. and Amoyel, M.** (2022). Cell-cycle exit and stem cell differentiation are coupled through regulation of mitochondrial activity in the Drosophila testis. *Cell Rep* **39**, 110774.

**Sanchez, C. G., Teixeira, F. K., Czech, B., Preall, J. B., Zamparini, A. L., Seifert, J. R., Malone, C. D., Hannon, G. J. and Lehmann, R.** (2016). Regulation of Ribosome Biogenesis and Protein Synthesis Controls Germline Stem Cell Differentiation. *Cell Stem Cell* **18**, 276-290.

**Senos Demarco, R., Uyemura, B. S. and Jones, D. L.** (2020). EGFR Signaling Stimulates Autophagy to Regulate Stem Cell Maintenance and Lipid Homeostasis in the Drosophila Testis. *Cell Rep* **30**, 1101-1116 e1105.

**Signer, R. A., Magee, J. A., Salic, A. and Morrison, S. J.** (2014). Haematopoietic stem cells require a highly regulated protein synthesis rate. *Nature* **509**, 49-54.

**Signer, R. A., Qi, L., Zhao, Z., Thompson, D., Sigova, A. A., Fan, Z. P., DeMartino, G. N., Young, R. A., Sonenberg, N. and Morrison, S. J.** (2016). The rate of protein synthesis in hematopoietic stem cells is limited partly by 4E-BPs. *Genes Dev* **30**, 1698-1703.

**Venken, K. J., Schulze, K. L., Haelterman, N. A., Pan, H., He, Y., Evans-Holm, M., Carlson, J. W., Levis, R. W., Spradling, A. C., Hoskins, R. A., et al.** (2011). MiMIC: a highly versatile transposon insertion resource for engineering Drosophila melanogaster genes. *Nature methods* **8**, 737-743.

**Xu, R., Li, J., Zhao, H., Kong, R., Wei, M., Shi, L., Bai, G. and Li, Z.** (2018). Self-restrained regulation of stem cell niche activity by niche components in the Drosophila testis. *Dev Biol* **439**, 42-51.

**Yuen, A. C., Hillion, K. H., Wang, R. and Amoyel, M.** (2021). Germ cells commit somatic stem cells to differentiation following priming by PI3K/Tor activity in the Drosophila testis. *PLoS Genet* **17**, e1009609.

**Zhang, S., Pan, C., Lv, X., Wu, W., Chen, H., Wu, W., Wu, H., Zhang, L. and Zhao, Y.** (2017). Repression of Abd-B by Polycomb is critical for cell identity maintenance in adult Drosophila testis. *Sci Rep* **7**, 5101.

**Zismanov, V., Chichkov, V., Colangelo, V., Jamet, S., Wang, S., Syme, A., Koromilas, A. E. and Crist, C.** (2016). Phosphorylation of eIF2alpha Is a Translational Control Mechanism Regulating Muscle Stem Cell Quiescence and Self-Renewal. *Cell Stem Cell* **18**, 79-90.
