# Supplementary material for: Signals from the niche promote distinct modes of translation initiation to control stem cell differentiation and renewal in the Drosophila testis
Source: PLoS Biol. 2025 Mar 11;23(3):e3003049. doi: 10.1371/journal.pbio.3003049 (PMC12136000; doi:10.1371/journal.pbio.3003049)
Supplement: Attachment — Submitted filename: Response_to_reviewers_auresp_3.docx [file pbio.3003049.s015.docx]

We thank the reviewers and the editors for their careful consideration of our manuscript and their constructive comments. We have made some changes to the text as suggested by the reviewers, as well as modified the title. We amended the suggested title slightly to “"Signals from the niche promote distinct modes of translation initiation to control stem cell differentiation and renewal in the Drosophila testis” as we felt that “in Drosophila” implied that our findings applied to all stem cells in Drosophila. In addition, we noticed a typo in Figure S2 where we had included incorrect N values in two of the graphs (swapping round some values accidentally). These have been corrected in the revised version. Furthermore, we have provided two additional data files with the raw data: S1_data includes all quantifications used in the graphs in each figure, showing all the data points and, if normalised, how the normalisation was done, and S2_data shows the original western blot images and explains how they were processed.

We hope that the manuscript will now be judged to be suitable for publication and we thank you once again for your constructive criticism and support through this process.


------------------------------------------------------------------------

Reviewer remarks:


Reviewer #1: I appreciate the author made useful improvements to the manuscript.

Two major outstanding issues remain:

1. It seems very important to show that eIF3d is phosphorylated since the phosphorylation state and eIF4F interaction is one of the major findings of the manuscript. Can authors use proteomics to detect modification or at least a proxy of CK2-eIF3d interaction?

We agree our data have not shown direct phosphorylation in vivo, however, the technical challenges posed by assessing this without an antibody in a rare population of cells are huge and would require many years of work to overcome. We have added a sentence in the discussion to raise the caveat that we have not shown direct phosphorylation, which reads: “Although we did not directly assess the phosphorylation status of eIF3d1 in CySCs in vivo, due to technical limitations, our data strongly suggest that eIF3d1 is indeed phosphorylated by CkII in the testis”.

2. It is still difficult to understand how a very subtle ~10-25% reduction (depending on TBB or CX-5011 treatment) in non-phosphorylated eIF3d-eIF4F interaction leads to changes in CySC self renewal.

Treatment with CX-5011 resulted in a 31% reduction in eIF4G1 pulldown and a 40% reduction in eIF4A pulldown (the figures for TBB are 12% and 18% respectively). We agree this is not an all-or-nothing effect, and it is indeed intriguing to speculate how this change could result in cell fate changes. We can only hypothesise currently that a change in translation efficiency might be enough to drive translation of a factor that can promote differentiation even at lower levels.

The expression of the endogenous UTR-containing Eya construct is a helpful addition. Can the authors could use a variation of this experiment to at least prove that this small change leads to gene-specific changes by expressing a heterologous UTR-containing Eya construct and showing this is now resistant to CKII inhibition and changes to eIF3d phosphorylation? This would allow authors to better link a small shift in interaction to specific translation control.

We agree this would be an informative experiment. Given the time frame, we believe this lies outside the scope of the current manuscript, but we do indeed plan to conduct these and similar experiments to follow up on this work. In the long term, we hope to build a comprehensive understanding of how CkII promotes fate changes through translation, and what specific transcripts are regulated in this manner.

In addition, in Figure 6G, equal eIF4A is being pulled down with the RAP2A control as with eIF3d WT. This is also seen in Figure R1 with RAP2A versus eIF3D DD for eIF4A1, since the IP is not cleaner it is difficult to consider the influence of eIF3d phosphorylation of the eIF4F and eIF3D interaction.

We have indeed observed a small but reproducible non-specific interaction between eIF4A and the beads we used for pulldown. However, pulldown is always higher when eIF3d is transfected, including in the figure we showed in the last response to reviewers with TBB instead of CX-5011.

Minor comment:
Authors state: "We show that phospho-dead eIF3d1, but not a form of eIF3d1 that lacks the mRNA 5' cap- binding domain, acts as a dominant-negative for CySC self-renewal, and moreover, that only full length or phospho-mimetic eIF3d1 can rescue self-renewal upon knockdown of CkII."

I could not find this data with eIF3d1 without the 5' cap binding domain - does the author mean a mutant where the cap-binding function is blocked (eIF3d1-helix11)? If so this is also incorrectly described in the results.

We apologise for this mistake, we did indeed mean that the cap-binding function is blocked, not that the domain was deleted. We have corrected this in the text.


Reviewer #3: The authors have done an overall good job for the revision and addressed most of the previous questions. However, I do have an issue for the FISH data shown in the new Figure 8: Given the high background in particular in the area containing cyst cells, it is really hard to tell whether these are real signals or which cells these "dots" belong to. In many cases, it looks like from the germ cells instead of cyst cells to me. The data quality in Figure 3 using similar approach gave out better images. I think this needs to be addressed, in particular if these are representative images used for quantification.

We thank the reviewer for their positive assessment of our work overall.

Regarding their comment, we do not think that the staining in Figure 8 is background, as CkII is expressed in all cells of the testis, including somatic CySCs and cyst cells, as well as the germline. Thus, it is different from the *in situ* data in Figure 3, where expression was restricted to cyst cells and appears “cleaner”. We note that expression increases in spermatocytes (towards the bottom of each image), again suggesting a real expression pattern and not uniform background. Finally, we show decreased expression in *Stat92e^ts^* mutants, further supporting the idea that the staining we show is indeed real expression.


Reviewer #4: The revised version of the manuscript by Wang and colleagues has greatly improved. We congratulate the authors for their constructive answers to our comments and for performing additional important experiments. We don't have any additional comment, except praising the authors for this nice work.

We thank the reviewer for their constructive comments that have helped improve our work and for their kind words.
